# Cancer-Associated Substitutions in RNA Recognition Motifs of PUF60 and U2AF65 Reveal Residues Required for Correct Folding and 3′ Splice-Site Selection

**DOI:** 10.3390/cancers12071865

**Published:** 2020-07-11

**Authors:** Jana Kralovicova, Ivana Borovska, Monika Kubickova, Peter J. Lukavsky, Igor Vorechovsky

**Affiliations:** 1 Faculty of Medicine, University of Southampton, Southampton SO16 6YD, UK; jakr@soton.ac.uk; 2 Institute of Molecular Physiology and Genetics, Center of Biosciences, Slovak Academy of Sciences, 840 05 Bratislava, Slovakia; ivana.sevcikova@savba.sk; 3 CEITEC, Masaryk University, 625 00 Brno, Czech Republic; monika.kubickova@ceitec.muni.cz (M.K.); lukavsky@ceitec.muni.cz (P.J.L.)

**Keywords:** Functional genomics, pre-mRNA splicing, 3′ splice site, mRNA, lariat branch point, *PUF60*, *U2AF2*, *SF3B4*, gel shift assay, differential scanning fluorimetry, cancer, leukemia, driver mutation, exon inclusion

## Abstract

U2AF65 (*U2AF2*) and PUF60 (*PUF60*) are splicing factors important for recruitment of the U2 small nuclear ribonucleoprotein to lariat branch points and selection of 3′ splice sites (3′ss). Both proteins preferentially bind uridine-rich sequences upstream of 3′ss via their RNA recognition motifs (RRMs). Here, we examined 36 RRM substitutions reported in cancer patients to identify variants that alter 3′ss selection, RNA binding and protein properties. Employing PUF60- and U2AF65-dependent 3′ss previously identified by RNA-seq of depleted cells, we found that 43% (10/23) and 15% (2/13) of independent RRM mutations in U2AF65 and PUF60, respectively, conferred splicing defects. At least three RRM mutations increased skipping of internal *U2AF2* (~9%, 2/23) or *PUF60* (~8%, 1/13) exons, indicating that cancer-associated RRM mutations can have both *cis*- and *trans*-acting effects on splicing. We also report residues required for correct folding/stability of each protein and map functional RRM substitutions on to existing high-resolution structures of U2AF65 and PUF60. These results identify new RRM residues critical for 3′ss selection and provide relatively simple tools to detect clonal RRM mutations that enhance the mRNA isoform diversity.

## 1. Introduction

Next-generation sequencing studies of cancer cells have identified mutations affecting important protein domains in RNA processing factors involved in 3′ splice site (3′ss) selection, first described in myelodysplasias [1]. In several genes, most notably *SF3B1* (also known as SF3b155) and *U2AF1* (U2AF35), the mutations are restricted to missense variants that encode just a few amino acids [1,2,3]. These alterations can directly impair contacts with precursor messenger RNAs (pre-mRNAs), as exemplified by substitutions in U2AF35 zinc-finger domains [4], or may act indirectly. For example, cancer-associated mutations in the HEAT domains of *SF3B1* [1] were proposed to lead to changes in the positioning of other RNA-binding proteins, including the 65 kilodalton (kD) subunit of the U2 small nuclear ribonucleoprotein auxiliary factor (U2AF65), ultimately altering interactions with pre-mRNAs [5] and the selection of lariat branch points and 3′ss [6,7,8]. Apart from *SF3B1* and *U2AF1*, clonal mutations have been found in genes encoding other 3′ss recognition factors, including *U2AF2* and *PUF60*, but at lower frequencies [1,9]. The two proteins bind highly variable and uridine-rich 3′ss consensus motifs known as the polypyrimidine tracts (PPTs), cooperatively recruiting the U2 small nuclear ribonucleoprotein by simultaneously binding to SF3b155 [10,11,12,13,14]. *U2AF2* and *PUF60* mutations in cancer cells are less restricted than those in *SF3B1* or *U2AF1* [1]; nevertheless, a previous study mapped recurrent mutations to U2AF65-RNA interfaces [15]. However, the functional fraction of PUF60/U2AF65 mutations in their RNA recognition motifs (RRMs) is unknown and their impact on protein properties and cancer initiation is not fully understood.

RRM-containing proteins make use of sophisticated strategies to interact with a large repertoire of single-stranded sequences with a considerable range of affinities [16]. Both U2AF65 and PUF60 preferentially bind uridine (U)-rich RNA sequences at PPTs via their RRMs to recruit U2 small nuclear ribonucleoprotein particles to the branch site [10,11,14,17,18]. Both U2AF65 and PUF60 contain central tandem RRMs and C-terminal U2AF-homology domains (UHMs), but PUF60 lacks the N-terminal arginine/serine-rich (RS) domain and the UHM-ligand motif (ULM) [10,12,18]. Through global identification of PUF60-regulated exons, we have recently characterized splicing abnormalities in cells overexpressing PUF60 that contained germ-line RRM missense mutations [14] found in Verheij syndrome (also known as or PUF60 deficiency, PD, or 8q24.3 microdeletion syndrome) [19,20]. Interestingly, PD-associated RRM substitutions generated a spectrum of splicing defects that could be seen with single tested exons [14]. PUF60 is required for cell viability, proliferation and migration in vitro and is often overexpressed in (pre-)malignant tissues [21,22], suggesting that the heterogeneity of missense RRM mutations in cancer cells might influence 3′ss/branch site recognition and expand mRNA isoform diversity. Nevertheless, this hypothesis has not been systematically tested and functional RRM substitutions remain unidentified. 

In the present study, we employed PUF60- and/or U2AF65-dependent splicing reporters and biochemical assays to discover cancer-associated missense mutations in PUF60 and U2AF65 RRMs that impair protein function. We have found that RRM substitutions can cause splicing defects of their target 3′ss (*trans*) or their own transcripts (*cis*), which may further enhance the mRNA diversity of cancer cells. We also identified cancer substitutions that altered protein folding/stability and reduced RNA binding to multiple 3′ss in vitro. These results validate new and relatively simple tools for the detection of functional U2AF65 and PUF60 substitutions and reveal strong clonal candidates that may drive cancer initiation or progression. They should also facilitate the design of structure-guided RRM variants that could selectively alleviate splicing defects in cancer cells.

## 2. Materials and Methods

### 2.1. Plasmid Preparations

Plasmid pET28a-PUF60-His for expression of human PUF60 (transcript ENST00000526683) in *E. coli* was described previously [23]. PUF60 was subcloned into BamHI/XhoI sites of pcDNA3.1/*myc*-His A (Invitrogen) in-frame with the *myc* tag at the C-terminus. The WT U2AF65 (a generous gift of Professor Andrew Berglund, University at Albany, New York, NY, USA) was subcloned into the same vector using BamHI/XhoI digests. 

Conserved missense RRM1 and RRM2 mutations for testing were selected from the highly curated and publicly available catalogue of somatic mutations in cancer (COSMIC) database (v.83) [9]. Mutated PUF60 and U2AF65 plasmids were prepared by overlap extension PCR using mutagenic primers shown in Table S1. PCR products were digested with BamHI/XhoI and ligated into pcDNA3.1/*myc*-His A using a T4 DNA ligase (Promega). Minigenes for testing inclusion levels of *U2AF2* and *PUF60* exons with RRM mutations were prepared using primers listed in Table S2. Sequences of all reporters are shown in Figure S1. All plasmids were propagated in *E. coli* DH5α; plasmid DNA was extracted using the GeneJET Plasmid Miniprep Kit (Thermo Fisher, Waltham, MA, USA). Constructs were validated by Sanger sequencing. Each construct lacked undesired mutations, except for U2AF65 G326E, which contained an extra missense mutation in the same RRM. 

### 2.2. Cell Cultures and Transfections

Validated plasmid DNA (200 ng) was transiently co-transfected with the reporter plasmid (80 ng) and 30 ng of the pcDNA3.1-EGFP into human embryonal kidney (HEK) 293 cells obtained from the Leibniz-Institute DSMZ-German Collection of Microorganisms and Cell Cultures (cat. ACC305). Transfections were carried out in 24-well plates using jetPRIME (Polyplus) according to manufacturer’s recommendations. Cells were harvested 48 h after transfection for RNA and protein preparations. PUF60 depletion was performed as described previously [14].

### 2.3. Detection of Spliced Products

Total RNA was extracted using TRI-reagent (Ambion, Austin, TX, USA), treated with DNase I (Promega, Chilworth, UK) and transcribed using the Moloney murine leukaemia virus (MMLV) reverse transcriptase (RT, Promega) and primer d(T)_20_ according to the manufacturers’ recommendations. RT-PCR reactions were performed using vector-specific primers or a gene- and vector-specific combination (Table S2), as described previously in detail [14,24,25]. Uncut *OGDH* products were amplified with primers PL4 and 35F, whereas *OGDH* products digested with HinfI, which cut only exon 4b, were amplified with primers PL4 and 35m-amplF (Table S2). RT-PCR amplifications were for 29 cycles to maintain approximately linear relationship between the RNA input and output signals. PCR products were separated on agarose or polyacrylamide gels and stained with GelRed (Biotium, Fremont, CA, USA). Signal intensity of the spliced products was measured as described [26]. 

### 2.4. Utilization of Cryptic 3′ss of UBE2F Exon 5 in Human Endogenous Transcripts

We employed a panel of total RNAs isolated from human tissues, each containing a pool from several different donors (FirstChoice; Life Technologies, Carlsbad, CA, USA). The first strand cDNAs were prepared using d(T)_20_ and MMLV RT. RT-PCR reactions were carried out with primers UBE2F-cr and UBE2F-R or UBE2F-F and UBE2F-R (Table S2). Apart from the cryptic 3′ss of exon 5, the former pair also amplified a cryptic exon in intron 4 in a tissue-specific manner. The identity of each RNA product was confirmed by Sanger sequencing.

### 2.5. Immunoblotting

Cells were washed with PBS and lysed in the RIPA buffer (150 mM NaCl, 1% NP-40, 0.5% deoxycholate, 0.1% SDS, 50 mM Tris, pH 8.0). Protein lysates were loaded onto 10% SDS-PAGE, transferred on to nitrocellulose membranes and incubated with antibodies against c-*myc* (PLA0001, Sigma), GFP (ab290, Abcam) and the His tag (ab1187-100, Abcam). Secondary antibodies were purchased from Thermo Fisher (#31458). Proteins were detected using the Pierce ECL Western Blotting Substrate (Thermo Fisher) according to the manufacturer’s instructions. 

### 2.6. Protein Expression and Purification

Constructs for the expression of recombinant WT and mutated PUF60 were created by inserting validated BamHI/XhoI fragments into the pET-28 vector with 2 × His-lipoyl-TEV site. The proteins were expressed in BL21-CodonPlus (DE3)-RIL competent cells (Agilent Technologies). The cells were grown to OD 0.8 at 37 °C. Protein expression was induced with 1 mM IPTG overnight at 16 °C. Cell pellets were dissolved in a 1 M NaCl, 20 mM HEPES, pH 7.5, 10% glycerol (w/v), 30 mM imidazole and 3.6 mM β-mercaptoethanol buffer containing the cOmplete™, an EDTA-free Protease Inhibitor Cocktail (Roche), and were lysed using EmulsiFlex-C3 (Avestin Europe GmbH). After centrifugation, the supernatants were loaded onto the Ni^2+^-charged HiTrap IMAC HP columns (GE Healthcare Life Sciences) through 0.45 mm filters and eluted in a buffer containing 0.5 M NaCl, 20 mM HEPES (pH 7.5), 10% glycerol (w/v), 300 mM imidazole and 3.6 mM β-mercaptoethanol. His-tags were removed by overnight cleavage with the in-house TEV protease in a buffer containing 500 mM NaCl, 20 mM HEPES, 10% glycerol (w/v) and 3.6 mM β-mercaptoethanol. The TEV protease was removed using the charged HiTrap IMAC HP column. For long-term storage, proteins were dialyzed against a 50 mM potassium phosphate buffer (pH 6.8) containing 300 mM NaCl, 10% glycerol (w/v) and 3.6 mM β-mercaptoethanol, freezed in liquid nitrogen and stored at −80 °C.

U2AF65 was expressed as a GST fusion protein using pGEX6P-U2AF65 (a generous gift of Professor Andrew Berglund). Mutated plasmids carrying G154V, E162K, E207G and G319V were prepared by inserting BamHI/XhoI fragments into pGEX6P-GST and were again validated by sequencing. The recombinant U2AF65 proteins were produced in BL21 (DE3) pLysS competent cells (Promega). Cells were grown at 37 °C; protein expression was induced with 1 mM IPTG. The pellets were dissolved in 50 mM Tris-HCl (pH 8.0), 200 mM NaCl, 1 mM EDTA and 1 mM DTT containing cOmplete™, an EDTA-free Protease Inhibitor Cocktail (Roche, Basel, Switzerland), and were sonicated using SONOPULS GM Mini20 (Bandelin Electronic, Berlin, Germany). The recombinant proteins were purified from supernatant using Glutathione Sepharose^®^ 4 Fast Flow (GE Healthcare, Chicago, IL, USA), washed four times with PBS and eluted in 50 mM Tris-HCl (pH 8.0), containing 20 mM reduced glutathione. Purified proteins were dialyzed against 25 mM Tris-HCl (pH 7.5), 300 mM NaCl, 15% glycerol (w/v), 1 mM DTT, using Slide-A-Lyzer™ G2 Dialysis Cassettes (Thermo Fisher).

### 2.7. Electrophoretic Mobility Shift Assay (EMSA)

Gel shift assays were carried out essentially as described [23], but with biotinylated RNAs (Table 1). Briefly, WT and mutated proteins were incubated with the indicated oligoribonucleotides in a binding buffer containing 5 mM MgCl_2_, 0.25 μg/μL heparin, 40 mM Tris (pH 8.0), 0.01% Triton and 1 mM DTT at room temperature for 20 min. RNA/protein complexes were separated on 6% native polyacrylamide gels run in 0.5× TBE at 4 °C, blotted onto nylon membranes (GE Healthcare) and crosslinked on a 254-nm UV transilluminator for 60 s. The products were detected with LightShift^®^ Chemiluminescent EMSA Kit (Thermo Fisher) according to manufacturer’s recommendations. Signal intensity was measured with the Amersham Imager 600 (GE Healthcare). The data were plotted (SigmaPlot, v.11) as a function of protein concentration and fitted to the Hill equation (B = B_max_*(L^n^/(L^n^+K_d_^n^)) where B_max_ is a bound fraction of RNA (B) at the saturating protein concentration L and n is the Hill coefficient) to determine dissociation constants (K_d_). 

### 2.8. Differential Scanning Fluorimetry (DSF)

Recombinant proteins were incubated in the 96-well thin-wall PCR plate (Bio-Rad, Hercules, CA, USA) for 10 min at room temperature. PUF60 reactions contained 9 μM of the protein, 70 mM potassium phosphate (pH 7.5), 130 mM NaCl, 5 mM MgCl_2_, 1.5 mM β-mercaptoethanol and 4% glycerol. U2AF65 reactions contained 8 μM of the protein, 50 mM Tris (pH 7.5), 150 mM NaCl, 5 mM MgCl_2_, 0.5 mM DTT and 7.5 % glycerol. After incubation, SYPRO Orange dye (Sigma-Aldrich, St. Louis, MO, USA) was added and plates were sealed with Microseal B adhesive sealer (Bio-Rad). After brief centrifugation, samples were heated on the CFX96 Real-Time System (Bio-Rad) from 25 to 95 °C at a rate of 1 °C per minute. The reporter was HEX (λ_ex_ 515–535 nm/λ_em_ 560–580 nm). The midpoint calculations were determined from the indicated melting curves using the CFX Manager (v. 3.1, Bio-Rad).

### 2.9. Solubility and Stability Predictions

Solubility profiles with WT and mutant PUF60/U2AF65 were generated by CamSol [27]. The effect of RRM substitutions on protein stability was predicted by the mutation cutoff scanning matrix [28].

## 3. Results

### 3.1. Selection of Functional Assays for Cancer-associated Substitutions in PUF60 and U2AF65 RRMs 

**Figure 1:**
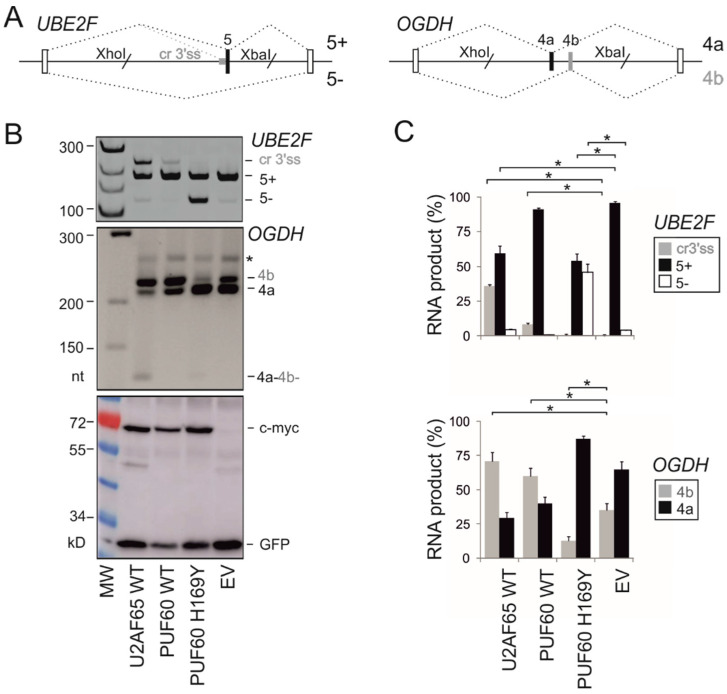
Altered exon usage of PUF60-dependent exons by U2AF65. (**A**) Schematics of PUF60-dependent hybrid reporter constructs. Their wild-type sequences are in Figure S1. Exons are shown as boxes, introns as horizontal lines, spliced products as dotted diagonal lines above and below the pre-mRNA. Designation of the spliced products is to the right. Grey horizontal rectangle denotes the (T)_n_G repeat of the canonical 3′ss PPT [14], slashes denote restriction sites used for cloning and empty boxes represent heterologous exons, as described [25]. (**B**) *UBE2F* and *OGDH* splicing patterns in cells overexpressing the indicated U-binding proteins. MW, molecular weight; EV, empty vector. PUF60 H169Y is a germline substitution reported in the Verheij syndrome [19]. Asterisk denotes slow-mobility heteroduplex DNA formed by annealing of homologous exons 4a and 4b. Immunoblotting is shown in the lower panel; antibodies are to the right. (**C**) Relative abundance of RNA products shown in panel B. Error bars: SDs of two independent transfection experiments. Asterisks denote significant differences (*p* values < 0.05, unpaired two-tailed t-tests) between EV and indicated constructs.

To test the response of PUF60-dependent 3′ss [14] to U2AF, we first transiently co-transfected two splicing reporters with constructs expressing the wild-type (WT) U2AF65 into HEK293 cells (Figure 1A). The first reporter was derived from *UBE2F* exon 5, which requires PUF60 for full inclusion in the mRNA [14]. The second construct contained mutually exclusive *OGDH* exons 4a/4b where PUF60 depletion repressed exon 4b [14]. The U2AF65 overexpression activated a cryptic 3′ss upstream of the canonical 3′ss of *UBE2F* exon 5 and induced exon skipping (Figure 1B,C). In *OGDH*, overexpression of either PUF60 or U2AF65 promoted exon 4b, yet individual depletion of the two proteins showed opposite effects of depletion and overexpression on exon 4a/4b usage only for PUF60 [14]. A PD-associated mutation in PUF60 RRM (H169Y) [19] failed to activate the cryptic 3′ss of *UBE2F* exon 5 as the WT protein while inducing exon 5 skipping and was unable to promote *OGDH* exon 4b. Together, these results indicated that the *UBE2F* reporter should be informative for testing splicing outcomes of cancer-associated RRM mutations in both proteins while *OGDH* is suitable for examining PUF60 variants.

### 3.2. PUF60 and U2AF65 RRM Substitutions that Alter 3′ss Usage

**Figure 2:**
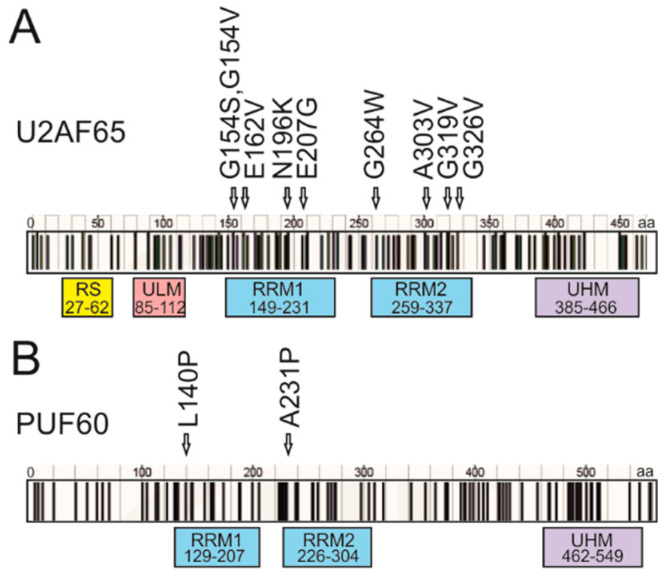
Distribution of cancer-associated substitutions in U2AF65 and PUF60. (**A**,**B**) Distribution of somatic missense mutations in the COSMIC database (v. 83). Their frequency is not shown since the COSMIC data could not be corrected for redundant entries, such as repeat samples from the same patient. PUF60 and U2AF65 substitutions that affected pre-mRNA splicing of our tested reporters are shown by arrows above RRM domains. (A) U2AF65. (B) PUF60. RRM alignments with tested substitutions are in Figure S2.

To identify functional RRM substitutions previously reported in cancer cells, we first compiled a list of conserved RRM1 and RRM2 missense mutations (Figure 2, Table S1) from the COSMIC database [9]. COSMIC data showed that missense mutations in cancer patients were reported at relatively high frequencies, both in PUF60 (63%) and in U2AF65 (71%). However, it remains uncertain if this may be due to their recurrent occurrences in RRM1 of U2AF65, as suggested by Glasser et al. [15], or in other functional domains (Figure 2). Accumulation of missense mutations in RRMs might be present in other 3′ss recognition factors (Figures S2 and S3A). The clustering of mutations within conserved domains would support their importance in cancer initiation or progression and the role of *U2AF2* or *PUF60* as oncogenes as opposed to tumor suppressors [29,30].

**Figure 3:**
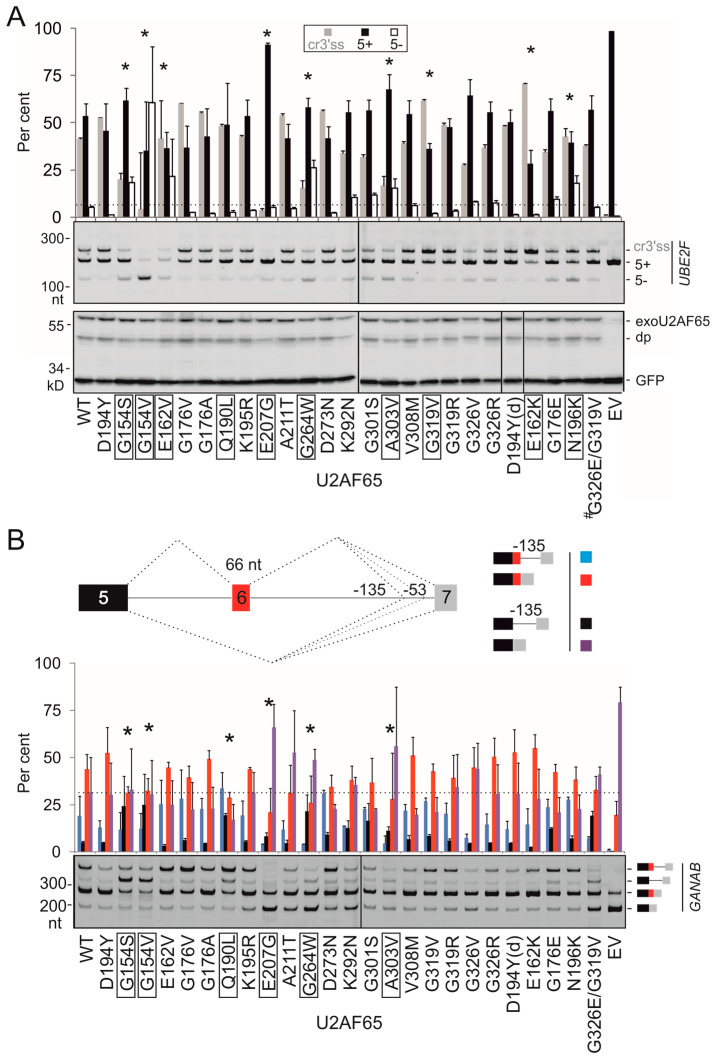
Splicing activities of cancer-associated U2AF65 RRM substitutions. (**A**) *UBE2F* splicing activities (upper panel) and immunoblotting of lysates from transfected HEK293 cells with anti-c-*myc* antibodies, which detect exogenously expressed protein (lower panel). The relative usage of *UBE2F* products is quantified at the top. WT, wild-type; EV, empty vector; ^#^, double mutant. The bar chart represents the average of four independent transfections ± SEM. One-way analysis of variance was performed with Dunnett’s multiple comparison post-hoc t-tests (STAT200). Asterisks denote significant deviations of mutated plasmids from the WT (*p* < 0.05). Functional substitutions are boxed. dp, degradation product. Dotted horizontal line indicates the level of exon skipping of the WT construct. (**B**) *GANAB* splicing activities of U2AF65 RRM substitutions. The reporter is shown in the upper panel; RNA products are to the right. The lower panel shows the relative usage of the spliced products. Error bars are SEMs of two independent transfections. Analysis of variance was performed with Dunnett’s multiple comparison post-hoc t-tests (STAT200, Biosoft). Dotted horizontal line denotes exon 6 skipping in the WT. Cryptic 3′ss at position -53 (Figure S1) was used at <2% and was disregarded.

**Figure 4:**
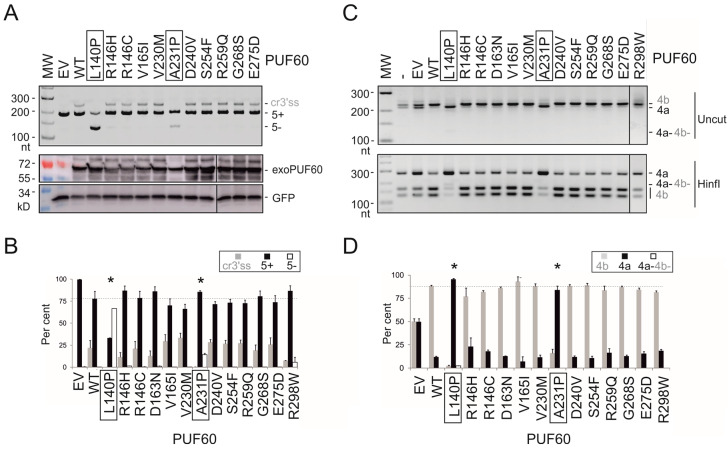
Splicing activities of cancer-associated PUF60 RRM substitutions. (**A**) *UBE2F* splicing pattern induced by PUF60 expression constructs with the indicated RRM substitutions (upper panel). A corresponding immunoblot is in the lower panel. (**B**) Relative usage of RNA products shown in panel A. Bar charts represent the means of duplicate transfections ± SEM. Dotted line indicates canonical transcripts induced by the WT protein. Amino acid substitutions with significant deviations from the WT (*p* < 0.05) are denoted by asterisks at the top and are boxed at the bottom. One-way analysis of variance was performed with Dunnett’s multiple comparison post-hoc t-tests. (**C**) Mutually exclusive usage of *OGDH* exons in HEK293 cells expressing the indicated constructs. For better resolution, undigested products (upper panel) were also digested with HinfI, which only cleaves exon 4b (lower panel). (**D**) Measurements of mRNA isoforms shown in panel C. Bar charts represent the means of duplicate transfections ± SEM. Dotted line indicates 4b transcripts induced by the WT protein. Amino acid substitutions with significant deviations from the WT (*p* < 0.05) are denoted by asterisks at the top and are boxed at the bottom. Analysis of variance was followed by Dunnett’s post-hoc t-tests.

Next, we prepared plasmids expressing U2AF65/PUF60 with mutated RRMs and measured the relative abundance of RNA products that employed competing 3′ss of *UBE2F* exon 5 upon transient co-transfections into HEK293 cells (Figure 3 and Figure 4). Of 23 different U2AF65 mutations tested, 10 (43%) altered 3′ss usage as compared to the WT, despite exhibiting similar overexpression levels on immunoblots. Of 13 tested PUF60 substitutions, 2 (15%) failed to promote the cryptic 3′ss and induced exon 5 skipping instead (L140P and A231P, Figure 4A,B). A231P was the only substitution that diminished PUF60 expression (Figure 4A, lower panel). In contrast to PUF60 and U2AF65, expression of the WT SF3B4 in HEK293 cells did not noticeably activate cryptic 3′ss in *UBE2F* (Figure S3B).

Next, we extended our splicing assays with mutated U2AF65 and PUF60 to *GANAB* and *OGDH* reporters, respectively (Figure 3B and Figure 4C,D). *GANAB* exon 6 is promoted in cells lacking U2AF65 [13,24], possibly via direct binding of U2AF65 to this exon [13]. We found that most U2AF65 substitutions affected splicing of both reporters, including highly conserved G154S/G154V in RRM1 or G264W in RRM2; however, we also observed reporter-specific effects (discussed below). In contrast, both deleterious PUF60 substitutions were concordant. 

We also introduced substitution D194Y in the U2AF65 RRM1 since the mutation is located at the same alignment position as the germline substitution PUF60 H169Y (Figure S2), which caused PD [19]. We observed no aberrant splicing with *UBE2F* and *GANAB* pre-mRNAs (Figure 3). This residue has not sustained any missense changes in cancer cells to date, but a synonymous mutation (c.582C>T; D194D) was found in cancer cells [9].

**Figure 5:**
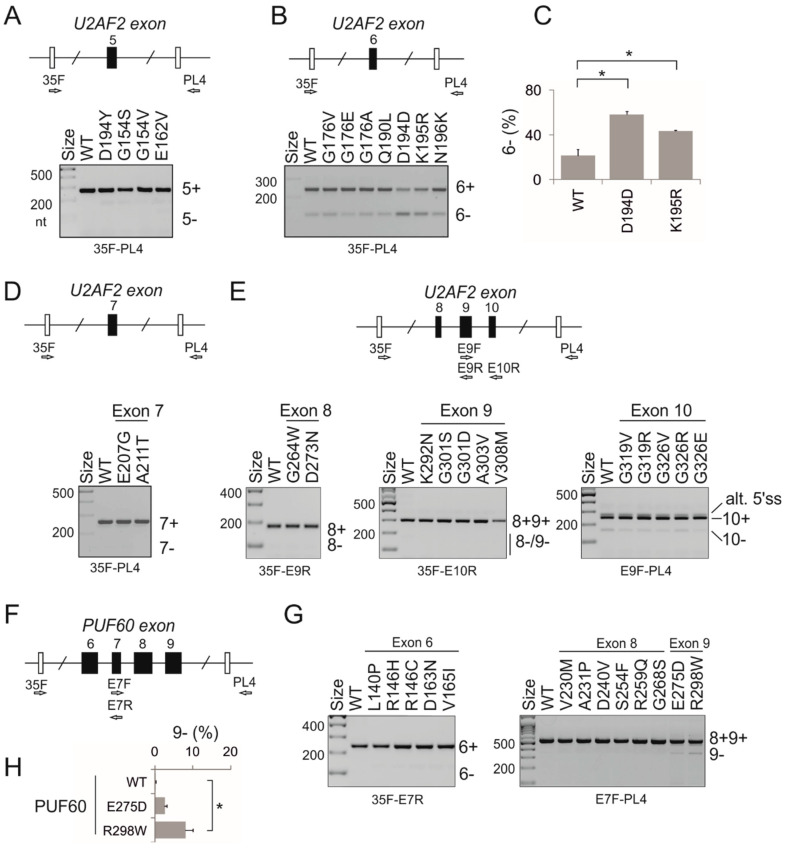
Identification of cancer-associated *U2AF2* and *PUF60* missense mutations that alter exon inclusion in *cis*. (**A**–**E**) *U2AF2*, (**F**–**H**) *PUF60*. Minigene constructs are schematically depicted above exon-specific panels. Tested exons are denoted by black rectangles and numbered. Terminal minigene exons are shown as open rectangles. RT-PCR primers (Table S2) are denoted by arrows and cloning sites by a slash. RNA products are shown to the right; +, exon inclusion; −, exon exclusion in the mRNA. Exon inclusion levels are in panels C (*U2AF2*) and H (*PUF60*). Error bars are SDs (n-method) of two transfection experiments. Asterisks denote significant differences between the WT and mutants (*p* < 0.05; one-way analysis variance with Dunnett’s multiple comparison post-hoc tests). Alt. 5′ss (panel E) is an alternative GC 5′ss of *U2AF2* exon 10, which is used by endogenous HEK293 transcripts at <25% [24].

Because missense, nonsense or synonymous mutations may alter *cis*-elements required for accurate exon selection [31,32,33], we examined splicing of a series of reporters containing the same RRM mutations in *U2AF2* (Figure 5A–E) or *PUF60* (Figure 5F–H) exons. We found that at least one missense (K195R) and one synonymous (D194D) mutation in *U2AF2* (Figure 5A–C) and one missense mutation in *PUF60* (R298W) significantly increased exon skipping.

Together, these results show that sensitive 3′ss previously identified by RNA-Seq in cells lacking PUF60 can provide useful substrates for rapid identification of functional cancer-associated RRM mutations not only in PUF60, but also in a cooperating protein. They also demonstrate that distinct RRM substitutions can confer a spectrum of splicing outcomes even for a single intron or exon. Conversely, the same substitutions could impair distinct types of alternative splicing, as illustrated by U2AF65 G154V, which activated cryptic 3′ss in *GANAB* primary transcripts while inducing *UBE2F* exon 5 skipping (Figure 3). Finally, our results demonstrate that RRM mutations can have both *cis*- and *trans*-acting effects on splicing, further expanding the diversity of mature transcripts (Figure 3, Figure 4 and Figure 5).

### 3.3. Functional Consequences of Cancer-Associated RRM Substitutions in U2AF65 and PUF60

**Figure 6:**
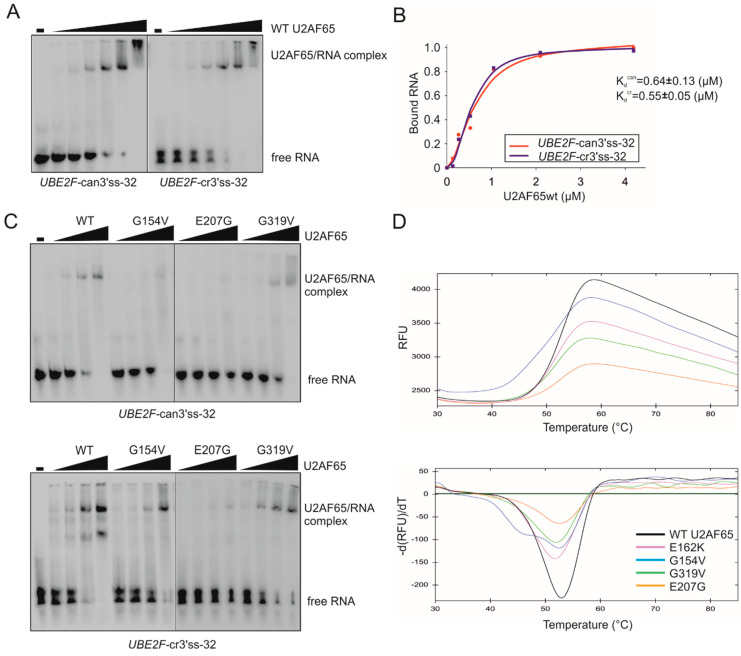
Cancer-associated U2AF65 RRM substitutions impair RNA binding and denaturation profiles. (**A**) In vitro binding of WT U2AF65 to PPTs of canonical and cryptic 3′ss of *UBE2F* exon 5, Concentrations of recombinant U2AF65 proteins were 0.13, 0.26, 0.52, 1.05, 2.1 and 4.2 µM. Sequences of RNA probes are in Table 1. (**B**) Fractions of bound RNA probes and K_d_ estimates. (**C**) Impaired binding of representative mutated U2AF65 to each PPT. Concentrations of recombinant U2AF65 were 0.26, 0.52, 1.05 and 2.1 µM. Concentration of biotin-labeled RNAs (bottom) was 17 nM. (**D**) Thermal denaturation profiles of U2AF65 RRM mutants determined by DSF with SYPRO Orange as a thermofluor. Melting curves are in the upper panel and melting peaks are in the lower panel.

To explore mechanisms underlying the observed *trans*-acting effects (Figure 3 and Figure 4), we employed EMSA and DSF assays with purified recombinant proteins and their putative U-rich targets at sensitive 3′ss. EMSA with the WT U2AF65 and biotin-labeled oligoribonucleotides derived from the PPT of canonical (can) or cryptic (cr) 3′ss of *UBE2F* exon 5 (Table 1) revealed similar binding affinities for each probe (Figure 6A,B). As compared to the WT, RNA binding of proteins carrying a subset of functional RRM substitutions was impaired to a varying degree (Figure 6C). DSF profiles (Figure 6D) also revealed variable defects. For G154V, DSF showed two heat absorption peaks, consistent with a biphasic unfolding transition, potentially reflecting a disunited domain unfolding [34], and with the reduced EMSA signals (Figure 6C). This mutation diminished the use of both competing 3′ss, i.e., induced exon 5 skipping (Figure 3A), which was reminiscent of the effect of PUF60 H169Y (Figure 1). In contrast, G319V and E162K showed less severe splicing alterations (Figure 3), a smaller decrease in melting temperatures (T_m_) in DSF profiles (Figure 6D) and still detectable RNA binding in vitro (Figure 6C, Figure S4). Chemiluminiscent EMSA with E162K showed similar binding affinities to the two *UBE2F* probes (Figure S4).

**Figure 7:**
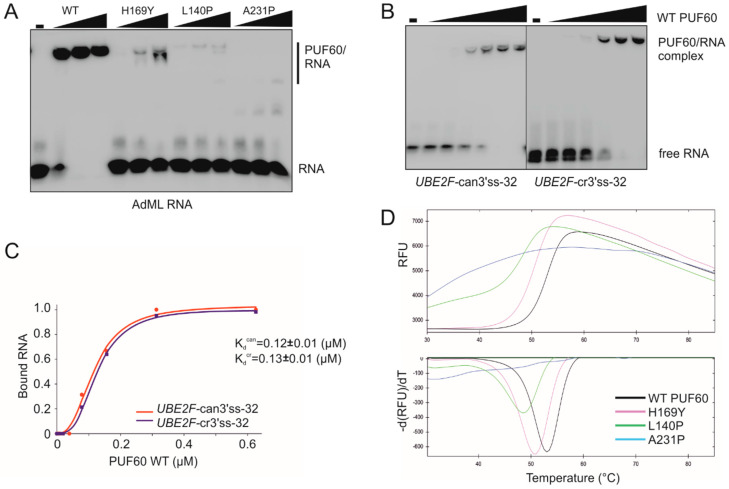
Cancer-associated PUF60 RRM substitutions impair RNA binding and denaturation profiles. (**A**) Impaired binding of PUF60 with RRM substitutions to the AdML RNA. Concentrations of recombinant proteins were 0.14, 0.42 and 1.25 µM. The AdML oligoribonucleotide sequence is in Table 1. (**B**) In vitro binding of WT PUF60 to canonical and cryptic 3′ss of *UBE2F* exon 5. Concentrations of recombinant PUF60 were 19.5, 39, 78, 156, 312 and 626 nM. RNA probes (bottom) are shown in Table 1. Biotin-labeled RNAs in panels A and B were at 15 nM. (**C**) Graphical representation of bound fractions with mean K_d_ values (±SD). The Hill coefficients were 2.6 ± 0.3 (can) and 3.0 ± 0.2 (cr). (**D**) Thermal denaturation profiles of PUF60 RRM mutants determined by DSF.

EMSA with WT PUF60 confirmed strong binding to a positive control derived from AdML [11] and showed reduced binding of each cancer-associated RRM mutation that produced *trans*-acting defects (Figure 7A; Table 1). Their binding to *UBE2F* oligoribonucleotides was also impaired (Figure S5). H169Y showed an intermediate binding to AdML (Figure 7A). Although binding affinities of the WT PUF60 to PPTs of canonical and cryptic 3′ss of *UBE2F* exon 5 were similar, H169Y appeared to bind to the former with a higher affinity (Figure 7B,C and Figure S6A,B), which would be consistent with a failure to activate the cryptic 3′ss (Figure 1). Among tested germline mutations, H169Y also produced the highest amount of RNA products lacking *UBE2F* exon 5 [14].

The hierarchy in AdML EMSA signals for functional PUF60 mutants (WT>H169Y>L140P>A231P) was mirrored by DSF, with T_m_ of H169Y reduced by ~2 °C and T_m_ of L140P by ~4 °C (Figure 7D). A231P showed flat DSF melting curves indicative of impaired folding, consistent with the diminished signal on immunoblots (Figure 4A). Unlike the WT, each of the three substitutions failed to induce the *UBE2F* cryptic 3′ss, but the ratio of *UBE2F* 5+ and 5− transcripts was consistently higher for A231P than L140P. Both L140P and A231P were resistant to TEV protease cleavage (Figure S7) and were predicted to alter solubility and protein stability by CamSol [27] and the mutation cutoff scanning matrix [28], respectively (Figure S8). Reduced solubility was also predicted for H169Y (Figure S8). 

Taken together, U2AF65 or PUF60 RRM substitutions that induce splicing abnormalities in *trans* not only alter binding affinities to RNAs derived from sensitive 3′ss but also impair biophysical properties of these proteins, including folding and thermodynamic stability.

### 3.4. Mapping of Cancer-Associated RRM Substitutions on to High-Resolution PUF60 and U2AF65 Structures 

**Figure 8:**
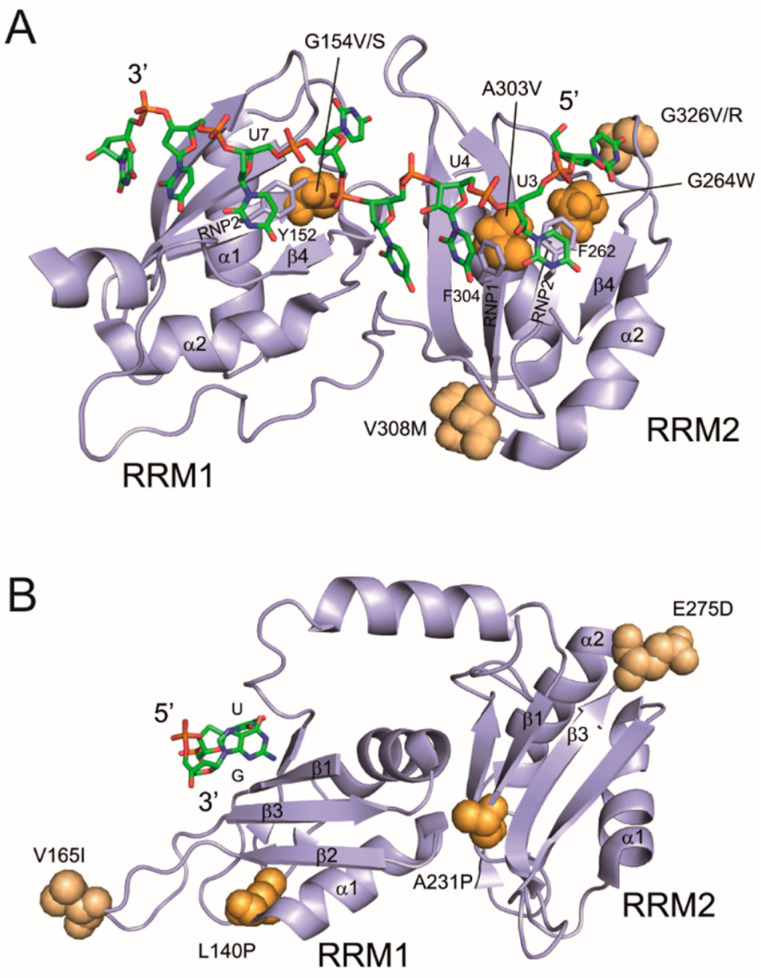
Cancer-associated substitutions mapped on to high-resolution RRM structures. (**A**) Crystal structure of U2AF65 tandem RRMs bound to poly(U) RNA (PDB entry 5EV1) [35]. (**B**) Crystal structure of PUF60 tandem RRMs bound to a modified AdML 3′ss analogue (PDB entry 5KW1, Crichlow G. V. et al., unpublished). (**A**,**B**) Mutated residues (labeled) are shown as space-filling atoms. Substitutions that affected 3′ss usage are in dark orange; substitutions that had no effect on minigene splicing are in light orange. Proteins (cartoon representation) are shown in purple. RNAs (stick representation) have carbon atoms in green, nitrogen atoms in blue, oxygen atoms in red and phosphorus atoms in orange.

Figure 8 shows examples of functional and neutral RRM substitutions in the context of previously determined structures of U2AF65 (5EV1) [35] and PUF60 (5KW1; Crichlow et al., unpublished) with short U-rich RNAs. In the U2AF65 structure (Figure 8A), the methyl group of A303 in the RNP1 motif makes a van der Waals contact with F304, which interacts with the uracil base of U4. Replacing this methyl group with the bulky side chain of valine produced splicing defects (Figure 3), most likely through altered positioning of F304, which would disrupt the uracil base stacking. Similarly, G264W in the RNP2 motif introduces the aromatic tryptophan side chain, which could interfere with stacking interactions of the uracil base of U3 with the top of the benzene ring of F262. Introducing the bulky tryptophan indole ring is likely to lead to a steric clash with F262 and interfere with U3 binding by RRM2. G154V, which reduced binding to *UBE2F* 3′ss and eliminated their recognition in cells (Figure 3 and Figure 6C), may have a similar effect on RRM1: Y152 (RNP2) stacks with the uracil base of U7, potentially rearranging the aromatic side chain and disrupting the stacking interaction. G154S, which produced less severe splicing defects (Figure 3), has a smaller side chain as compared to valine, potentially maintaining the U7-Y152 stacking interaction. Thus, these functional substitutions have a strong potential to disrupt interactions with poly(U) RNAs.

In contrast, a splicing-neutral V308M substitution (Figure 3) maintains a hydrophobic residue in the loop between RNP1 (β3) and helix α2 in RRM2 and does not bind to poly(U). Likewise, although G326V/R substitutions change a small glycine to bulky residues in the α2–β4 loop, they are solvent exposed and not in contact with the RNA. On the other hand, E162 is solvent exposed, away from the RNA binding site, and E162K did not appear to reduce binding to *UBE2F* RNAs, yet the substitution showed reproducible DSF and splicing alterations (Figure 3, Figure 6 and Figure 8A). 

In the PUF60 structure, functional mutations L140P in RRM1 and A231P in RRM2 (Figure 2, Figure 4 and Figure 7) reside in the β1–α1 loop, also far away from the RNA binding surface (Figure 8B). Nevertheless, they may affect RNA binding indirectly by disrupting local hydrophobic interactions, as suggested by DSF (Figure 7D). These substitutions would be predicted to destabilize the fold around the RNP modules. While U2AF65 mutations in the RNPs of RRM2 might directly interfere with poly(U) binding and change 3′ss usage, the substituted prolines in RRMs of PUF60 are likely to have an indirect, structural effect on the RNA binding surface, in turn altering the splicing outcome of PUF60-dependent 3′ss. By contrast, splicing-neutral V165I in RRM1 (β2–β3 loop) and E275D in RRM2 (β3–α2 loop) maintain a hydrophobic or acidic amino acid side chain, respectively, and are solvent-exposed without direct RNA contacts (Figure 8B).

Finally, the germline PUF60 H169Y substitution in RRM1 (β2–β3 loop) might also indirectly affect the RNP modules (Figure S9). Interestingly, the distance (2.9 Å) between the N^ε2^ atom of the imidazole ring and the backbone carbonyl atom of L140 of helix α1 suggests that the N^ε2^ nitrogen is protonated and forms a hydrogen bond with the L140 carbonyl group. This interaction could help stabilize the β2–β3 loop and in turn adjacent RNPs. H169Y would eliminate this hydrogen bond and insert a hydrophobic tyrosine ring into the hydrophobic core (I136, M160), altering the local RNP structure, consistent with the splicing defect (Figure 1B,C), lower T_m_ detected by DSF (Figure 7D) and lower binding affinities to *UBE2F* 3′ss (Figure S6). However, although the three functional PUF60 substitutions yielded a wide T_m_ spectrum, the T_m_ values were not completely mirrored by the 5+/5- ratios of *UBE2F* transcripts (*cf*. Figure 1, Figure 4 and Figure 7).

## 4. Discussion

We have experimentally characterized the impact of 36 cancer-associated RRM substitutions in PUF60 or U2AF65 on protein properties and splicing in *cis* and *trans*. Identification of mutations with *trans*-acting splicing defects was facilitated by sensitive 3′ss previously found by RNA-seq of cells depleted of PUF60 [14], highlighting the power of this method to identify exploitable targets of important splicing factors in the whole transcriptome. PUF60 and U2AF65 preferentially bind PPTs, interact with each other via their UHM and ULM and cooperate in 3′ss selection processes [10,11,12,14]. The transcriptome-wide identification of PUF60-dependent exons revealed that depletion of PUF60 or U2AF65 had often reciprocal effects on 3′ss usage [14] (Figure 1); hence, it is not surprising that functional defects of mutated proteins were detected by a single reporter containing 3′ss sensitive to both proteins. 

PUF60 and U2AF65 are required for accurate recognition of a substantive proportion of 3′ss [13,14]. For U2AF65, this fraction was estimated at ~58% [36] and ~88% [13], consistent with acting as a major PPT/3′ss recognizer. The detection rate of functional U2AF65 substitutions also tended to be higher than for PUF60 (43% vs 15%, *p* = 0.09, Fisher’s exact test; Figure 3 and Figure 4). Uridines are frequently recognized sequence-specifically by RRM proteins as compared to other ribonucleotides and their recognition is achieved through all nucleotide-binding pockets [16]. This suggests that U2AF65 and PUF60 RRM substitutions may turn out to be important contributors to the mRNA isoform diversity of cancer cells, acting both in *cis* and *trans* (Figure 3, Figure 4 and Figure 5). 

The two reporters were concordant for PUF60 variants (Figure 4), but not completely for U2AF65 (Figure 3). This may be attributable to the detection limits of our tests, but also to a higher sensitivity and/or lower variability in the relative abundance of *UBE2F* products, as compared to *GANAB*. Functional mutations in each protein may also potentially influence other RNA processing or gene expression steps: for example, U2AF is involved in 3′-end processing and PUF60 can also function as a transcriptional repressor [24,37]. Cancer RRM substitutions are likely to create a spectrum of splicing defects of a larger number of 3′ss than those revealed by our reporters. This spectrum was detected even with a single intron, suggesting that a binary classification of RRM substitutions as pathogenic or non-pathogenic is inappropriate.

The majority of RRM substitutions did not show any reduction in overexpression levels (Figure 3 and Figure 4) although we could not exclude minor differences in chemiluminescent signals. Neither PUF60 nor U2AF65 were identified among most dysregulated RBPs in a systematic expression screen across 15 cancer types [38]. Because UHM domain substitutions found in PD diminished PUF60 expression [14], cancer mutations in this domain could affect selection of PUF60-dependent 3′ss globally, potentially mimicking splicing abnormalities previously observed for the PUF60 knockdown [14]. Conversely, overexpression of PUF60 was observed in several cancer types, including hepatic and lung cancer where mRNA levels seem to reflect genomic gains at 8q24.3 around the *PUF60* locus and predict poor survival [22,39,40,41]. The PUF60 overexpression may facilitate detachment of cancer cells on a 3D matrix and their migration was promoted by PUF60 isoforms lacking exon 2/5 [41]. These isoforms produced splicing abnormalities of exogenous transcripts distinct from canonical PUF60 [14].

Our results should also motivate future studies aimed at characterizing the mRNA isoform diversity of PUF60 and U2AF65 RRM substitutions at the level of the whole transcriptome. Impaired alternative splicing of *UBE2F, GANAB* or *OGDH* in vivo may be important for cancer initiation or progression. UBE2F is involved in neddylation, which may alter protein function by conjugation of the ubiquitin-like protein NEDD8 to its targets. The process is catalyzed by a neddylation-activating enzyme E1, one of the two neddylation conjugating E2 enzymes (UBE2F and UBE2M), and by E3 ligases, including RBX1/2 [42,43]. UBE2F pairs with RBX2 to regulate cullin 5 whereas UBE2M pairs with RBX1 to mediate neddylation of cullins 1–4 [42]. UBE2F degradation is promoted by UBE2M, which keeps UBE2F in check by activating CRL3, ensuring a cross-talk between E2 and E3 [44]. Overexpression of *UBE2F* mRNA isoforms in cancer predicts poor survival and promotes tumor growth in vitro and in vivo while the *UBE2F* knockdown is inhibitory [45]. The 33-nt 5′ extension of *UBE2F* exon 5 binds both PUF60 and U2AF65 in vitro (Figure 1, Figure 6 and Figure 7) and is alternatively spliced, introducing extra 11 amino acids (GFFCFVLCFLI) in the peptide. Our RT-PCR assay with a panel of RNAs from human tissues and a forward primer across the cryptic 3′ss visualized a cryptic exon upstream, with the highest relative abundance of the cryptic 3′ss of exon 5 in brain and gonads (Figure S10). However, it remains to be seen whether the differences between tissues reflect tissue-specific expression levels [46], intron 4 variability among RNA donors, kinetics of intron removal or other factors. In the crystal structure model of the NEDD8 E1 ubiquitin-fold domain and the UBE2F core [42], the extra peptide would be inserted in an exposed disordered region between β2 and β3 sheets, potentially introducing a new interaction surface; however, the impact on neddylation, degradation of pro-apoptotic NOXA [45] or tumor growth remains to be characterized. Finally, splicing abnormalities of *UBE2* genes were common in lung carcinomas [47] and UBE2C was found among the most overexpressed transcripts [48].

*UBE2F* exon 5 is one of the most sensitive PUF60 targets in the whole transcriptome [14]. The 3′ss of exon 5 has an unusual upstream competitor separated by (U)_n_G repeats that bind both PUF60 and U2AF65 in vitro (Figure 6 and Figure 7, Figures S5 and S6). The two 3′ss employ distinct lariat branch sites; the downstream branch site is embedded in the cryptic 3′ss consensus [14]. Apart from repressing both 3′ss and exon skipping (G154V or N196K), RRM substitutions seemed to impair the fine balance between 3′ss in either direction (*cf*. G154S or E207G with E162K or G319V) (Figure 3). How exactly this putative bidirectional effect on selection of 3′ss competitors reflects impaired U2AF65 interactions with protein partners and specific RNA targets remains to be seen. This phenomenon evokes the co-existence of loss- and gain-of-function mutations in the same protein domains in cancer cells, as exemplified by activating and inactivating missense mutations in DNA-binding or kinase domains [29,30,49,50]. 

*GANAB* exon 6 encodes 22 amino acids that interrupt a unique disordered B1 subdomain of glucosidase IIα [51,52]. The first post-translationally modified residue in the peptide has been recently identified (DKIKNLF, the underlined lysine is ubiquitinylated) [53], but the precise role of the two isoforms is obscure. GANAB was among proteins most enriched in acidic exosomes implicated in melanoma progression, with high expression levels associated with poor prognosis [54]. It interacts with the short secreted isoform of ADAM12, which is overexpressed in many tumor types [55]. Knockdown of *GANAB* or *PRKCSH*, which encode the glucosidase II heterodimer, reduced Wnt3 secretion by 40%–50%, arguing for the role in Wnt signalling [56]. Finally, knockdown of *OGDH* was associated with profound growth defects in a subset of cancer cell lines, potentially providing a therapeutic target in a metabolically distinct subset of tumors [57], although the distinct function of mRNA isoforms was not addressed. 

Cancer mutations found in *U2AF2* were previously mapped to U2AF65 structures [15]. For example, N196K changes the RRM1 conformation to bind a uracil base [15] and leads to exon skipping (Figure 3). In contrast, U2AF65 G176 substitutions did not change the conformation to bind RNA [15] and induced no detectable splicing alterations (Figure 3). U2AF65 G301I reduced binding affinity to d(U)_20_, but the reduction was the smallest among tested mutations; G301I also packs against the sugar-phosphate backbone of uridine rather than interacting with the base [58]. In our assays, G301S did not show a splicing defect (Figure 3), but we cannot exclude that it would affect the use of other 3′ss. On the other hand, binding affinities of some mutants do not fully explain the splicing pattern. U2AF65 E207G showed a reduced EMSA signal with each *UBE2F* RNA probe (Figure 6C), but canonical 3′ss was still selected albeit less efficiently than for EV controls (Figure 3). In addition, binding of E162K to each probe (Figure S4) was similar, although the discrimination power of chemiluminescent EMSA may not disclose minor K_d_ changes. It is also worth mentioning that some cancer-associated PUF60 substitutions, such as V230M [59] or A231P [60], are at the same alignment positions that were previously implicated in side chain contacts between U2AF65 and U7 RNA (Figure S2) [58].

Finally, SF3B4 is a component of the SF3b complex required for branch site recognition [61], is the only SF3b protein with two RRMs adjacent to each other [62] and also has RNA binding preferences for GU-rich motifs, largely through RRM2 [63], yet our PPT reporters failed to respond (Figure S3). Whether this reflects a distinct set of SF3B4-dependent target 3′ss, rather than a lower expression of SF3B4 in our system, remains to be seen. In this respect, *KLF4* exon 3 has been recently proposed to be a prominent SF3B4 target, potentially acting in early-stage hepatocellular carcinomas [64]. Loss-of-function SF3B4 mutations in the germline lead to acrofacial dysostosis syndromes, including Nager/Rodriguez syndromes, and RNA-seq of mutated chondrocytes detected many splicing alterations [65], which may yield suitable reporter 3′ss for future testing of cancer variants.

## 5. Conclusions

Here we have exploited publicly available RNA-seq data to develop screening assays of cancer-associated PUF60/U2AF65 RRM substitutions to identify functional mutations. We found that besides RNA binding and 3′ss selection defects, a subset of RRM substitutions altered protein folding and stability and generated an unexpected mRNA diversity by promoting or repressing 3′ss competitors in their pre-mRNA targets. This diversity may be further enhanced by variable skipping of *PUF60* or *U2AF2* internal exons observed for a small fraction of RRM missense mutations. These results will facilitate functional characterization of RRM mutations in cancer cells in the future.

## Figures and Tables

**Table 1:** RNA probes for non-isotopic EMSA. ^1^ Asterisks denote biotin. ^2^ AdML RNA is derived from the adenovirus major late 3′ss and was previously shown to bind recom binant PUF60 [11]. The last nucleotide of each EMSA probe is at the first exon position.

Oligoribonucleotide	Sequence (5′-3′) ^1^
AdML ^2^	*UUCGUGCUGACCUGUCCCUUUUUUUUCCACAGC
*UBE2F*-can3′ss-32	*UUUUUUUGUUUUGUUUUGUGUUUUUUGAUAGA
*UBE2F*-cr3′ss-32	*CUUUUUGUUUCCUUUUUUUUUUAAUUUAAAGG

## Supplementary Materials

The following Tables and Figures are available online at https://www.mdpi.com/2072-6694/12/7/1865/s1; Table S1. Characterization of tested mutations; Table S2. Cloning and RT-PCR primers; Figure S1. Sequences of wild-type splicing reporter inserts; Figure S2. Alignment of human U2AF65, PUF60 and SF3B4 RRMs; Figure S3. Splicing pattern of PUF60-/U2AF65-dependent reporters in cells overexpressing SF3B4; Figure S4. U2AF65 E162K binding to oligoribonucleotides derived from canonical and cryptic 3′ss of *UBE2F* exon 5; Figure S5. Non-isotopic EMSA with WT and mutated PUF60 and RNA probes derived from competing 3′ss of *UBE2F* exon 5; Figure S6. Binding of PUF60 H169Y to *UBE2F*-derived oligoribonucleotides; Figure S7. PUF60 L140P and A231P induce protein destabilization and misfolding; Figure S8. Solubility and stability profiles of wild type and mutated PUF60 or U2AF65; Figure S9. H169Y in the crystal structure of PUF60 RRM1; Figure S10. Utilization of cryptic 3′ss of *UBE2F* exon 5 in human tissues.

## Abbreviations

AdML: adenovirus major late; COSMIC: catalog of somatic mutations in cancer; DSF: differential scanning fluorimetry; DTT: dithiothreitol; EDTA: ethylenediaminetetraacetic acid; EMSA: electrophoretic mobility shift assay; GFP: green fluorescent protein; GST: glutathione S transferase; HEK: human embryonal kidney; HEPES: 4-(2-hydroxyethyl)-1-piperazineethanesulfonic acid; HEX: hexachlorofluorescein; IMAC: immobilized metal affinity chromatography; IPTG: isopropyl β-D-1-thiogalactopyranoside; MMLV: Moloney murine leukemia virus; PUF60: poly(U)-binding splicing factor 60 kDa; RRM: RNA recognition motif; RT-PCR: reverse transcriptase polymerase chain reaction; SDS-PAGE: sodium dodecyl sulfate polyacrylamide gel electrophoresis; TEV: tobacco etch virus; U: uridine; U2AF65: 65-kD subunit of the auxiliary factor of U2 small nuclear ribonucleoprotein; UHM: U2AF-homology motif; ULM: UHM-ligand motif; WT: wild-type.
