# Supplementary material for: Cancer-Associated Substitutions in RNA Recognition Motifs of PUF60 and U2AF65 Reveal Residues Required for Correct Folding and 3′ Splice-Site Selection
Source: Cancers (Basel). 2020 Jul 11;12(7):1865. doi: 10.3390/cancers12071865 (PMC7408900; doi:10.3390/cancers12071865)
Supplement: Supplementary file 1 [file cancers-12-01865-s001.pdf]

SUPPLEMENTAL INFORMATION

Cancer-associated substitutions in RNA recognition motifs of PUF60 and U2AF65 reveal residues required for correct folding and 3' splice site selection

Jana Kralovicova[1,2], Ivana Borovska[2], Monika Kubickova[3], Peter J. Lukavsky[3], Igor Vorechovsky[1]

[1]University of Southampton
Faculty of Medicine
Southampton SO16 6YD
United Kingdom

[2]Slovak Academy of Sciences
Institute of Molecular Physiology and Genetics
840 05 Bratislava
Slovak Republic

[3]Masaryk University
CEITEC
625 00 Brno
Czech Republic

Table S1 Characterization of tested mutations

COSMIC mutation	Mutagenic primer[1]	Amino acid substitution	Domain	Secondary structure[2]	FATHHM score[3]	Malignancy[3]	Source[3]	Method[3]
PUF60-419T>C	CTACTATGAGCcGGGGGAGGACA	L140P	RRM1	not assigned	0.96	Colorectal cancer	(1)	WES
PUF60-437G>A	GGACACCATCCaCCAGGCCTTTG	R146H	RRM1	α helix	0.96	Breast cancer	(2)	WGS
PUF60-436C>T	AGGACACCATCtGCCAGGCCTTT	R146C	RRM1	α helix	0.97	Colon adenocarcinoma	(3)	WES
PUF60-487G>A	ACATGTCCTGGaACTCCGTCACC	D163N	RRM1	β sheet	0.96	Hairy cell leukaemia	(4)	WES
PUF60-493G>A	CCTGGGACTCCaTCACCATGAAG	V165I	RRM1	not assigned	0.99	Colon adenocarcinoma	ICGC(COAD-US)	WES
PUF60-688G>A	ACCGCATCTACaTGGCCTCTGTG	V230M	RRM2	β sheet	0.97	Malignant melanoma	(5)	WGS
PUF60-691G>C	GCATCTACGTGcCCTCTGTGCAC	A231P	RRM2	β sheet	0.98	Prostate cancer	(6)	WES
PUF60-719A>T	CCTCTCAGACGtTGACATCAAGA	D240V	RRM2	α helix	0.98	Prostate cancer	(7)	WES
PUF60-761_762CC>TT	GCAAGATCAAGTttTGCACACTGGCC	S254F	RRM2	β sheet	N.A.	Squamous cell carcinoma	(8)	WES
PUF60-776G>A	CACACTGGCCCaGGACCCCACAA	R259Q	RRM2	β sheet	0.98	Colon adenocarcinoma	(9)	WES
PUF60-802G>A	GCAAGCACAAGaGCTACGGCTTC	G268S	RRM2	β sheet	0.96	Colon adenocarcinoma	(9)	WES
PUF60-825G>T	ATTGAGTACGAtAAGGCCCAGTCG	E275D	RRM2	bend	0.96	Endometroid carcinoma	ICGC(UCEC-US)	WGS
PUF60-892C>T	GCCAGTACTTGtGGGTGGGCAAGG	R298W	RRM2	β sheet	0.96	Colon adenocarcinoma	(9)	WES
U2AF65-460G>A	GCCTCTACGTGaGCAACATCCCC	G154S	RRM1	β sheet	0.94	Myelodysplastic syndrome	(10)	TES
U2AF65-461G>T	CCTCTACGTGGtCAACATCCCCT	G154V	RRM1	β sheet	0.96	AML	(11)	TES
U2AF65-484G>A	TTGGCATCACTaAGGAGGCCATG	E162K	RRM1	α helix	0.95	Bladder carcinoma	(12)	WES
U2AF65-485A>T	TGGCATCACTGtGGAGGCCATGA	E162V	RRM1	α helix	0.97	Melanoma	ICGC(SKCM-US)	WES
U2AF65-527G>T	GATGCGCCTGGtGGGGCTGACCC	G176V	RRM1	α helix	0.94	Liver carcinoma	TCGA-CC-A7IH-01	WES
U2AF65-527G>A	GATGCGCCTGGaGGGGCTGACCC	G176E	RRM1	α helix	0.93	Lung adenocarcinoma	ICGC(LUAD-US)	WGS
U2AF65-527G>C	GATGCGCCTGGcGGGGCTGACCC	G176A	RRM1	α helix	0.93	Breast carcinoma	27135926	WES
U2AF65-569A>T	GTTGGCTGTGCtGATTAACCAGG	Q190L	RRM1	β sheet	0.98	CLL	(14)	WES
U2AF65-584A>G	TAACCAGGACAgGAATTTTGCCT	K195R	RRM1	turn	0.98	T-cell leukaemia	(15)	WES
U2AF65-588T>A	CAGGACAAGAAaTTTGCCTTTTT	N196K	RRM1	turn	0.80	AML	ICGC(LAML-KR)	WES
U2AF65-620A>G	CTCAGTGGACGgGACTACCCAGG	E207G	RRM1	α helix	0.98	Melanoma	ICGC(SKCM-US)	WES
U2AF65-631G>A	AGACTACCCAGaCTATGGCCTTT	A211T	RRM1	α helix	0.98	Prostate cancer cell line	23856246	WES
U2AF65-790G>T	AGCTGTTCATCtGGGGCTTACCC	G264W	RRM2	β sheet	0.99	Liver carcinoma	ICGC(LICA-CN)	WES
U2AF65-817G>A	ACCTGAACGATaACCAGGTCAAA	D273N	RRM2	α helix	0.99	Squamous cell carcinoma	(8)	WES
U2AF65-876G>T	AACCTGGTCAAtGACAGTGCCAC	K292N	RRM2	β sheet	0.89	Colorectal carcinoma	(17)	WES
U2AF65-901G>A	GGCTCTCCAAGaGCTACGCCTTC	G301S	RRM2	β sheet	0.97	Renal carcinoma	ICGC(KIRP-US)	WES
U2AF65-908C>T	CAAGGGCTACGtCTTCTGTGAGT	A303V	RRM2	β sheet	0.93	Melanoma	(18)	WES
U2AF65-922G>A	TCTGTGAGTACaTGGACATCAAC	V308M	RRM2	bend	0.96	Melanoma	(19)	WES
U2AF65-956G>T	GGCCATTGCGGtGCTGAACGGCA	G319V	RRM2	α helix	0.97	Cervix adenocarcinoma	ICGC(CESC-US)	WGS
U2AF65-955G>A	AGGCCATTGCGaGCTGAACGGC	G319R	RRM2	α helix	0.97	Squamous cell carcinoma	(20)	WES
U2AF65-977G>T	CATGCAGCTGGtGGATAAGAAGC	G326V	RRM2	turn	0.97	Glioma	TCGA-HT-A4DV-01	WES
U2AF65-976G>A	GCATGCAGCTGaGGGATAAGAAG	G326R	RRM2	turn	0.98	Lung adenocarcinoma	(21)	WES
U2AF65-977G>A	CATGCAGCTGGaGGATAAGAAGC	G326E	RRM2	turn	0.97	Pancreatic carc.	ICGC(PACA-IT)	WES
U2AF65 (PUF60 H169Y paralog)	AGATTAACCAGtACAAGAATTTTG	D194Y	RRM1	turn	N.A.	Germline PUF60 deficiency	(22)	WES

Legend: [1]Mutated positions are in lower case. [2] PDB accession numbers: 5KWQ (PUF60) and 2YH1 (U2AF65). [3]COSMIC database (v. 91). WES, whole exome sequencing; WGS, whole genome sequencing; TES, targeted exome sequencing.

Table S2 Cloning and RT-PCR primers

**Primer**	**Sequence (5'-3')**
**Expression plasmids**	
SF3B4-49F-*BamI*	ATTAGGATCCAGACGGCGGGATCTCTTT
SF3B4-49R-*XbaI*	ATTATCTAGACTGAGGGAGAGGGCCTCGAAGTG
U2AF2-R-*XhoI*	ATTACTCGAGCCAGAAGTCCCGGCGGTGAT
***PUF60* and *U2AF2* minigenes**	
PUF60-E6-F	ACCACTCGAGCTGCCTCCTGACCATCTGTC
PUF60-E9-R	ACCATCTAGACCCTGGTCTGGCTACTCAAG
U2AF2-E5-F	ACCACTCGAGCTTCTGAGGAGCAGCAGTT
U2AF2-E5-R	ACCATCTAGAGGGTTGCTGGAGGAGGTT
U2AF2-E6-F	ACCACTCGAGTGTTGTCATCATGCCCTCTG
U2AF2-E6-R	ACCATCTAGACTGGAAAAGGCCAAAGAGGT
U2AF2-E7-F	ACCACTCGAGCAGCACTTTGTCCCTCTTCC
U2AF2-E7-F	ACCATCTAGATTTGGTCTGATCCTGGCTCT
U2AF2-E8-E10-F	ACCACTCGAGTGTGAACTTTGTGCCTTTGG
U2AF2-E8-E10-F	ACCATCTAGACCAGTCTCCCTCTGCTCAAG
**RT-PCR**	
Vector primer PL3	GGGAGACCCAAGCTGGCTA
Vector primer PL4	AGTCGAGGCTGATCAGCGG
35F	CAGGTGCTCTCGGTTGCA
35m-amplF	GCTCGGATCCTACAGAGTCAA
U2AF2_E10R	CTCTGGACCAGCAGCTTCTT
U2AF2_E9F	AACCTGGTCAAGGACAGTGCCAC
U2AF2_E9R	GCACTGTCCTTGACCAGGTT
PUF60_E7F	GCCTTGGAGCAGATGAACTC
PUF60_E7R	AGCTTCGGGGACCTCATACT
UBE2F-F	GCATTTTCCTGATCCAAACAA
UBE2F-R	CCCTGTCTCTGTGATGTTGG
UBE2F-cr	GCTAACAGTAACCCCAGGATTT

Figure S1 Sequences of wild-type splicing reporter inserts

Full reporters are schematically shown in Figures 1A, 3B and 5. Exons are in upper case and intronic sequences are in lower case. Restriction sites are underlined. Tested exons (highlighted and in bold) and flanking intronic sequences are in black, *U2AF1* exons 2 and 4 and flanking intronic sequences are in grey. Conserved dinucleotides at cryptic (*UBE2F* and *GANAB*) or alternative (*U2AF2*) splice sites are in red. Plasmid mutations are shown in Table S1.

UBE2F

ggatcc**TACAGAGTCAACTGTTCATTTTATTTCAAAATTGGAGCATGTCGTCATGGAGACAGGTGCTCTCGGTTG**
CACAATAAACCGACGTTTAGCCAGgtttgtttgcctttttttcatgtaaattataaaaacttcatgttcttttca
aagacagttaatttctacatattaagcaagtcatttttctctcgtagttgtattttccatttgtgttcataagt
gtgttctttatttaaataatagtgaggcaggtgatcgacttccagtggaaggtctgagaccaccactccttgtt
tttatcattagagaaactttaaaaatcagtttttgatgtttgtgtgttggctgtgcctgatatttgctgccctgc
catcttccctacccctctgatagacatgggtccagaggggtggccgtgccagccttgggttagcctcctccag
gcaccagtggacacggagtgggctttctgctctttgctgtctccagacagtagaggtgatggagccaccttccaa
gttagcgtgattgtcaccaggcccatcatgctgctcgagctgttctgaaggctgcatcaagcaatcactgaggca
gtgattctgtcaggttcactgctgtgtccccaaggcctagaaccatgcccagcagaggttgggtgctcagtaagg
gtttgttggtgactgttagtcttgtgtcctccaattgacattctcttggggagaccagcagtaagcagataaaga
agtcacttaaggcatgagatgtgttcggagggttgtaggttccaggaacatggtaggatgcaaggaggccaggca
gatggccccagaaggatgtcccttcccttccctgagggtgtgctcagccccctgagaagacagacaggcaggtga
cagcatggagcccatttctgttcaggcaggggcaggacttccaccagtgtgtgtgcatttggtgatggaaccgag
gaactctgcaggcctgtttttaaactcctgattctctgtatttgccatatcctaacagagtgtttgctttttgtt
tcctttttttttttaatttaaaggatttttttgttttgttttgtgttttttgatag**ATGAGGGTTACTACCAGGGT**
GGAAAATTTCAGTTTGAAACTGAAGTTCCCGATGCGTACAACATGGTGgtgagtagcctgcgttgagcctgttgt
tttacttctcgggcggggctgcctgtggctgtggcccgccactggcacagggccctagcactcccctctgcgtgt
gtccatgtgtgactgagtactcacttcagtatttttgtgatgaatgtcactcatcagcagtatttagagtgttag
tagagaattccgtttattcagcaaatacttatgagtactatgtgccaggcagcctgcctgtgtattttctataac
tcttatctgttacagtctggcactgaagaaaaaaagcatttaataaaatgactgtagttttgttcatgatataac
accaaaattccagaagtctataaaatgtgtattttagaatttcagagaacagtgacttgtagtttaaactataag
taatttatacctatagtttcatgttaatccctcaaagatccttttagacaataattataccaaataccattacat
tatgtctttgctttggtggaacttctagataatctcgtgtgtgttactgtagcagtttgagtttaaacacaagag
ttcactcggttaacatgttctaaccagcaagatttctgttgtttgcag**GTGCCGTGAGCGATGTGGAGATGCAGG**
AACACTATGATGAGTTTTTTGAGgggccc

GANAB

gaattcg**GCTTTCTGTCTCTGGTCGTGATGAGAACAGTGTGGAGTTAACCATGGCTGAGGGACCCTACAAGATCA**
TCTTGACAGCACGGCCATTCCGCCTTGACCTACTAGAGGACCGAAGTCTTTTGCTTAGTGTCAATGCCCGAGGAC
TCTTGGAGTTTGAGCATCAGAGGGCCCCTAGGGTCTCgtgagtacaggggttgggactgcagggaacctagtgtg
aaagagcataggggtgttgtgaggtctggcacggggaggagacggtggtgggacagccaggtagggcctgggggc
tgggagaagctctctgtcagggagggtaggaaccagacccgggtgcggtttttccatttcctgtacagaccagg
aggcagtcacttttgtccctgagctgtaccctgggcatctctgggaggcctgcctgggtctgtgtctccttcttc
cccttctcacatcatcacatttcccagatttattttcttcgttttatttatttattttttttggtttctttttc
cccccatctctggaagtttgtcgactgaaactcactttatgtttctgtttttgtgttggttttgtgcctctttt
tccccttccctacccatctcctcctgccccag**TTTCTCGGATAAGGTTAATCTCACGCTTGGTAGCATATGGGAT**
AAGATCAAGAACCTTTTCTCTAGgtaaatccatggccaccggtactgtatctgttcctctgcccttacccattcc
tcactctagcctttgggggaggtgccccagacccttccagcctttcactcaccccactttcccctgctgtgtga
tgtctggtcctgttacctgttaccctggctgggagcccccagaagaaggaaggtgagatactgaaagtcaggtcc
ttcgggaatgtagggagcttattgctttgccaggggatggagagatgctgcctattgctggggttcctgactctc
agctgaggggcacctgaggttctgggcagagctacttgcccttgaaagcatttttttggcagaatctcttgaagtc
cctatgtatgtctgtagaggcttggtatagtatgacccacatgcctttgcaaagccattgtgtcaggccaggagg
ctgaggcccagggagagcacttggggccagaatctctcagtagaggcaacagagccaggattggcattcataacc
caggcccctgaatcattctgcctttctcttctgggatgttgacagaggctggtgttcaggcctgtgcctcctggt
ggcacttattcctcatttcctttctcatttctctccccatcctttgcaaacttatctgctcattgtgggttccc
tctccaagctttaacatttagtcccttcctccaatgccttcgttcctttggacccttggctctctattccccaac
ccctccttccactagcccctcgtccctgcccccctctggattggagcagacagctctcctaccttccag**GCAAGGA**
TCAAAAGACCCAGCTGAGGGCGATGGGGCCCAGCCTGAGGAAACACCCAGGATGGCGACAAGgcaagttctaga

OGDH

ggatcc**TACAGAGTCAACTGTTCATTTTATTTCAAAATTGGAGCATGTCGTCATGGAGACAGGTGCTCTCGGTTG**
CACAATAAACCGACGTTTAGCCAGgtttgtttgcctttttttcatgtaaattataaaaacttcatgttcttttca
aagacagttaatttctacatattaagcaagtcatttttctctcgtagttgtattttccatttgtgttcataagt
gtgttcttttatttaaataatagtgaggcaggtgatcgacttccagtggaaggtctgagaccaccactccttgtt
tttatcattagagaaactttaaaaatcagtttttgatgtttgtgtgttggctgtgcctgatatttgctgccctgc
catcttccctacccccctctgatagacatgggtccagagggggtggccgtgccagccttgggttagcctcctccag
gcaccagtggacacggagtgggctttctgctctttgctgtctccagacagtagaggtgatggagccaccttccaa
gttagcgtgattgtcaccaggcccatcatgctgctcgagtttggtttctggtggcatgggccatgggcaggtgtt
gtaatggggcaacataggggaggtcctgggtctctggcccttcatgccatggcctggcaagtaaggctctctccc
tggggcaagtgtattctgccttagaaaggagatgcatggagttttgtaaatgctatatgctttaaagtgttatcc
atgtggatatttgcataaggaggaagcccagtggtgtttctttatcactgcctgcacgcagttggcctcagtaac
aacttcagcctgatcttgcagtgacttaccagcagctgctcccttctcaaattgctagcaagaagttgtgaaaga
atcttaaattggaagggatttctttctccttttggtttttaaatagttcaaatcagatgctttaagtctgtggt
gtagcagggtttttttgttctgactactgtgtgctgacctactcagcagcagcaggaccagagccctcactcctc
acggggatatataataaaggcaaaccttctcttagtgagatgacctcttatctgcactcctccccagttaaggcc
aacacttaagccttcattttcctacttagaaaaatttttctttaaatttgatttattttaaattgaggtgggatc
tcactgtgttgcccaggttggcctcaaactcctaggctcaagagatcctcctacctcagcctcccaagtagctgg
aactacaggcatgtgccactgtacccagcaccttagggaaatgtttatgttctgagctgaggcaggcttcacatt
ggagtaagaattgcccagcatatctcatttctgtttatccatggaaactgtataaagatcacaagaaatggatcc
tttcagactcctaagtttatcataacctgagtttatctctttttttataaaagtacatttatgcacaatagtgctt
ttaaaataagttattccaagacttttaaaaactaatttgtcgtatgggtttattggtgctgtgttcctctttgg
aaaaggtttgcataacctgtgactcttttaaccctccccacagctcactggatctgcttaatgacttgcttgtgt
tattgcttccag**GTCAGGGGTCACCACATTGCAAAACTTGATCCTCTCGGAATTAGTTGTGTAAATTTTGATGAT**
GCTCCAGTAACTGTTTCTTCAAACGTGGgtgagaattaagctgtaaatgctaattttaatgtaattttacttttt
ttttaccccttccctctttttttttcttctgtccttttgtgtgtgtccttccctctcatcgttggccactcatag
ATACGAGGGCACCATGTAGCACAGCTGGACCCCCTGGGGATTTTGGATGCTGATCTGGACTCCTCCGTGCCCGCT
GACATTATCTCATCCACAGACAAACTTGgtgagggtctgagagcagtcagctgcgttgcttgagctcctctcgct
gtgccacgacctgtggtagccaggatgtccaggcaggagggaaaggctcttaagtttcctttcattctgatcact
tgaaatttatcggtattgtagccttagtgtgttttgggcaagttatttttatcttacttttttcagccagattgtc
ttaagtctcaaaatttgaaaataaaattttggacagaaaggtttgtggcacagggaaaaagcttgtccacctggg
gcaggtggatcagaagcacttcagagtatagctcttttggcttcaggttttcaaatgtcagtgcagcactgcctt
cagtgaggcatgtccatgaggccttgctcaggaactgctgacacagccatccttttctgaagcccagtgtggcct
gcccttgactggaggccatgtcccttaggggacgaattctagataatctcgtgtgtgttactgtagcagtttgag
tttaaacacaagagttcactcggttaacatgttctaaccagcaagatttctgttgtttgcag**GTGCCGTGAGCGA**
TGTGGAGATGCAGGAACACTATGATGAGTTTTTTGAGgggccc

***U2AF2* exon 5**

ggatcc**TACAGAGTCAACTGTTCATTTTATTTCAAAATTGGAGCATGTCGTCATGGAGACAGGTGCTCTCGGTTG**
CACAATAAACCGACGTTTAGCCAGgtttgtttgcctttttttcatgtaaattataaaaacttcatgttcttttca
aagacagttaatttctacatattaagcaagtcatttttctctcgtagttgtattttccatttgtgttcataagt
gtgttcttttatttaaataatagtgaggcaggtgatcgacttccagtggaaggtctgagaccaccactccttgtt
tttatcattagagaaactttaaaaatcagtttttgatgtttgtgtgttggctgtgcctgatatttgctgccctgc
catcttccctacccccctctgatagacatgggtccagagggggtggccgtgccagccttgggttagcctcctccag
gcaccagtggacacggagtgggctttctgctctttgctgtctccagacagtagaggtgatggagccaccttccaa
gttagcgtgattgtcaccaggcccatcatgctgctcgaggcttctgaggagcagcagttactgggtgggggcccc
gagggtggaaagagggttctggaaggtgctaagaccgagagcctgtaggagctttctgctgaggaggggaactcc
cagtgtgtggtgaggggtggtcactgagcattcccctgatggggttttatccggcttttattccctttgaag**CTG**
CGGGTCAGATTCCAGCCACTGCTCTTCTCCCCACCATGACCCCTGACGGTCTGGCTGTGACCCCAACGCCGGTGC
CCGTGGTCGGGAGCCAGATGACCAGACAAGCCCGGCGCCTCTACGTGGGCAACATCCCCTTTGGCATCACTGAGg
tactgccctcccctgcccccctaccctctcccttgtccctctaccccgttcctccccttccctccattccctttcc
ccaacctgcacgggtcagactctgctcctgcaagacccccggcccctcccagacggaccgatggagttgagcc
agccaggggccgggctgggggtggccctccctccctcctcttcccctctcccgtctcccctcccccaacctcct
ccagcaaccctctagataatctcgtgtgtgttactgtagcagtttgagtttaaacacaagagttcactcggttaa
catgttctaaccagcaagatttctgttgtttgcag**GTGCCGTGAGCGATGTGGAGATGCAGGAACACTATGATGA**
GTTTTTTGAGgggccc

***U2AF2* exon 6**

ggatcc**TACAGAGTCAACTGTTCATTTTATTTCAAAATTGGAGCATGTCGTCATGGAGACAGGTGCTCTCGGTTG**
CACAATAAACCGACGTTTAGCCAGgtttgtttgcctttttttcatgtaaattataaaaacttcatgttcttttca
aagacagttaatttctacatattaagcaagtcatttttctctcgtagttgtattttccatttgtgttcataagt
gtgttcttttatttaaataatagtgaggcaggtgatcgacttccagtggaaggtctgagaccaccactccttgtt
tttatcattagagaaactttaaaaatcagtttttgatgtttgtgtgttggctgtgcctgatatttgctgccctgc
catcttccctacccccctctgatagacatgggtccagagggggtggccgtgccagccttgggttagcctcctccag
gcaccagtggacacggagtgggctttctgctctttgctgtctccagacagtagaggtgatggagccaccttccaa
gttagcgtgattgtcaccaggcccatcatgctgctcgagtgttgtcatcatgccctctgcctttttcttgaacaat
cttctcctttcccctgatttttttggggcccctgtggctgtcccccaacgcctgtccatcgcctgcccttggccgt
ctcttgctactgcttcctccccatttctctcttgcttctgttcttactattgacttctacacccccacccatat
tccactgttccttcccccccctcctccctcccctcatccctctccccttacccccaaacccctctgtgtctccctc
tctcacccttcccctcctctctccacctcccttcccccgcccccccccttgtctcctattccctctgcag**GAGGC**
CATGATGGATTTCTTCAACGCCCAGATGCGCCTGGGGGGGCTGACCCAGGCCCCTGGCAACCCAGTGTTGGCTGT
GCAGATTAACCAGGACAAGAATTTTGCCTTTTTGGAGgtgagctgggggagtgagtgaggtccaggaaacgtgtg
tgatgtgagggcccagcccttaggctgatgttgtgtggagctgccttgtgctgtttccaccaggccctctgcagc
ctcttctccacccggtcgctgaagtgagccctgttatagcattacagatctgatcctactttctgccttccattt
gctttctgtgtgacctgagaggcctgtttggcctctgtagacctctttggccttttccagtctagataatctcgt
gtgtgttactgtagcagtttgagtttaaacacaagagttcactcggttaacatgttctaaccagcaagatttctg
ttgtttgcag**GTGCCGTGAGCGATGTGGAGATGCAGGAACACTATGATGAGTTTTTTGAG**gggccc

***U2AF2* exon 7**

ggatcc**TACAGAGTCAACTGTTCATTTTATTTCAAAATTGGAGCATGTCGTCATGGAGACAGGTGCTCTCGGTTG**
CACAATAAACCGACGTTTAGCCAGgtttgtttgcctttttttcatgtaaattataaaaacttcatgttcttttca
aagacagttaatttctacatattaagcaagtcatttttctctcgtagttgtattttccatttgtgttcataagt
gtgttcttttatttaaataatagtgaggcaggtgatcgacttccagtggaaggtctgagaccaccactccttgtt
tttatcattagagaaactttaaaaatcagtttttgatgtttgtgtgttggctgtgcctgatatttgctgccctgc
catcttccctacccccctctgatagacatgggtccagagggggtggccgtgccagccttgggttagcctcctccag
gcaccagtggacacggagtgggctttctgctctttgctgtctccagacagtagaggtgatggagccaccttccaa
gttagcgtgattgtcaccaggcccatcatgctgctcgagcagcactttgtccctcttccgtgtcatcacagcctg
caattgtgtgtttgcctctgtgtttgtgttgggtgcctggtgtttttaatggtcgagtgtgagctcctggcagca
gggacatgcgtgtctgttgtactcccagtgcccagcctggggcctggcatgttgtatttgttcacatgagtgaaa
ggaaagaggaaatcccaatcctggaacactaggggctgtactagtccctgacccccatccctcaccactcctttc
tctttcattcag**TTCCGCTCAGTGGACGAGACTACCCAGGCTATGGCCTTTGATGGCATCATCTTCCAGGGCCAG**
TCACTAAAGATCCGCAGGCCTCACGACTACCAGCCGCTTCCTGGCATGTCAGAGAACCCCTCCGTCTATGTGCCT

Ggtgagtgggggatccattaagggcccctttctcccccaatcctgttcccatggcctgtccatcccttcagccca
gctggagtggcttaggaagttgtgtaagtactgtggaagataggtgccttttccctgcttaccctaagtctctgt
gagtggggtgctctcctgctggtagagaaggtgcctagggccgggagaatgagctgtcacctgctgaggacacac
agcttgtacgtggcagagccaggatcagaccaaatctagataatctcgtgtgtgttactgtagcagtttgagttt
aaacacaagagttcactcggttaacatgttctaaccagcaagatttctgttgtttgcag**GTGCCGTGAGCGATGT**
GGAGATGCAGGAACACTATGATGAGTTTTTTGAGgggccc

***U2AF2* exons 8-10**

ggatcc**TACAGAGTCAACTGTTCATTTTATTTCAAAATTGGAGCATGTCGTCATGGAGACAGGTGCTCTCGGTTG**
CACAATAAACCGACGTTTAGCCAGgtttgtttgcctttttttcatgtaaattataaaaacttcatgttcttttca
aagacagttaatttctacatattaagcaagtcatttttctctcgtagttgtattttccatttgtgttcataagt
gtgttcttttatttaaataatagtgaggcaggtgatcgacttccagtggaaggtctgagaccaccactccttgtt
tttatcattagagaaactttaaaaatcagtttttgatgtttgtgtgttggctgtgcctgatatttgctgccctgc
catcttccctacccctctgatagacatgggtccagaggggtggccgtgccagccttgggttagcctcctccag
gcaccagtggacacggagtgggctttctgctctttgctgtctccagacagtagaggtgatggagccaccttccaa
gttagcgtgattgtcaccaggcccatcatgctgctcgagtgtgaactttgtgcctttggagcgttctggaggatg
ttctagtttgaggttcccctgaggctgggcagatttgggagacatggtgcgttctccccgaggagcctctgtgtg
ccactgcccaggaccctcactggtcagataaggctggggtggggtgggcacgtggcgacccctccctcgtcagat
caggcaggaagtgttctctttggcaattgaggagctcgccgtagactgtccaggttttgggagataacctggtac
tggaattgaagtcctcctcttctctacccatag**GGGTTGTGTCCACTGTGGTCCCCGACTCTGCCCACAAGCTGT**
TCATCGGGGGCTTACCCAACTACCTGAACGATGACCAGgtaacttccctgcctccctccagacccgtccccccac
cccgccccacctcatcccagccctgatggactctcggctactgcag**GTCAAAGAGCTGCTGACATCCTTTGGGCC**
CCTCAAGGCCTTCAACCTGGTCAAGGACAGTGCCACGGGGCTCTCCAAGGGCTACGCCTTCTGTGAGTACGTGGA
CATCAACGTCACGGATCAGgtgagtccccggtcgctggccgctgccgcgtctgtccttccctgccctgcgctgtt
gccaagccatggtctcccctcctcaggggacggggcgggaggcggccaacctgaggcagtgccctgcgtgtgggc
tcgtccctgtcccatggcgttggctttttccaggctcttaatcccctttgccttcccctcttcccccaccaatgc
cccggcttggggtaggtgtcgggctcctggtccctgaccccacctctccccgcacccccccgacccctcatcctc
caaacacag**GCCATTGCGGGGCTGAACGGCATGCAGCTGGGGGATAAGAAGCTGCTGGTCCAGAGGGCGAGTGTG**
GGAGCCAAGAATGCCACGCTGgtgagccccccggcacgtcatcttccattggcttgagggaccctgggggtggga
ggggctggctagtaggggacaagtgttcctgatctttctcccagctttcatgggaaggcatttgggggcattca
gtgcagtgttggggagtcgggctttaggtgttggaagtggagagatggcctttcccctgggggggcacgtgaggg
gcctaggcttgagcagagggagactggtctagataatctcgtgtgtgttactgtagcagtttgagtttaaacaca
agagttcactcggttaacatgttctaaccagcaagatttctgttgtttgcag**GTGCCGTGAGCGATGTGGAGATG**
CAGGAACACTATGATGAGTTTTTTGAGgggccc

***PUF60* exons 6-9**

ggatcc**TACAGAGTCAACTGTTCATTTTATTTCAAAATTGGAGCATGTCGTCATGGAGACAGGTGCTCTCGGTTG**
CACAATAAACCGACGTTTAGCCAGgtttgtttgcctttttttcatgtaaattataaaaacttcatgttcttttca
aagacagttaatttctacatattaagcaagtcatttttctctcgtagttgtattttccatttgtgttcataagt
gtgttcttttatttaaataatagtgaggcaggtgatcgacttccagtggaaggtctgagaccaccactccttgtt
tttatcattagagaaactttaaaaatcagtttttgatgtttgtgtgttggctgtgcctgatatttgctgccctgc
catcttccctacccctctgatagacatgggtccagaggggtggccgtgccagccttgggttagcctcctccag
gcaccagtggacacggagtgggctttctgctctttgctgtctccagacagtagaggtgatggagccaccttccaa
gttagcgtgattgtcaccaggcccatcatgctgctcgagctgcctcctgaccatctgtcggctttgctgccgcag
tctgcctgggaccttggcgggctggggtgcgtcctgtcctgccgctgggcctggccttccttctttgggtggcgg
ggtcctgcgacccccacactatgactgcagggatggagcccgcacctccctgtcatgtggggagccctccccaca
gcagggctgggtgggtgggccaggaggggagtgggctgcccagcttcctgccgcccctgacgagccagcggttct
ctcccctctgtcctgctgcag**ATGGCGGCTCAGCGGCAGCGGGCGCTGGCCATCATGTGCCGCGTCTACGTGGGC**
TCTATCTACTATGAGCTGGGGGAGGACACCATCCGCCAGGCCTTTGCCCCCTTTGGCCCCATCAAGAGCATCGAC
ATGTCCTGGGACTCCGTCACCATGAAGCACAAGgtcagcaggcttggtccgccccggccacttcgggctcgcccc
cacccctgggctcgcgcagcctgacaggtgtgtccctgtgtctag**GGCTTTGCCTTCGTGGAGTATGAGGTCCCC**
GAAGCTGCACAGCTGGCCTTGGAGCAGATGAACTCGGTGATGCTGGGGGGCAGGAACATCAAGgtgaggcaggga
gccaaggcctcgatcccgcaggcgtgcagggctgcccctccacggaagccttttgtggccgggcctgggttgacc
ggtctttccatctcaccgcctcttccccag**GTGGGCAGACCCAGCAACATAGGGCAGGCCCAGCCCATCATAGAC**
CAGTTGGCTGAGGAGGCACGGGCCTTCAACCGCATCTACGTGGCCTCTGTGCACCAGGACCTCTCAGACGATGAC
ATCAAGAGCGTGTTTGAGGCCTTTGGCAAGATCAAGTCCTGCACACTGGCCCGGGACCCCACAACTGGCAAGCAA
AGGGCTACGGCTTCATTGgtgagctggggtggctgaggcgggatgggggccacctgaggctggggctggccctgc
tcactgctgctcctgcccacag**AGTACGAGAAGGCCCAGTCGTCCCAAGATGCTGTGTCTTCCATGAACCTCTTT**
GACCTGGGTGGCCAGTACTTGCGGGTGGGCAAGGCTGTCACACCGCCCATGCCCCTACTCACACCAGCCACGCCT
GGAGGCCTCCCACCTGCCGCTGCTGTGGCAGCTGCTGCAGCCACTGCCAAGATCACAGCTCAGgtgagggcccac
acagctgtcggcttgagggtgggcgggctggcccctgattccttggagactgattcaaggtggtcttgagtagcc
agaccagggtctagataatctcgtgtgtgttactgtagcagtttgagtttaaacacaagagttcactcggttaac
atgttctaaccagcaagatttctgttgtttgcag**GTGCCGTGAGCGATGTGGAGATGCAGGAACACTATGATGAG**
TTTTTTGAGgggccc

Figure S2 Alignment of human U2AF65, PUF60 and SF3B4 RRMs

Residues that sustained missense mutations in cancer (23) are in red; residues tested in this work are highlighted in yellow. Substitution D231V that selectively increased affinity for uridines and discriminated against purines in a short synthetic PPT-derived DNA (24) is highlighted in grey. A conserved aromatic residue that recognized a guanine in *syn* (24) is in magenta. Residues mutated in the Verheij syndrome (22,25) are highlighted in green. rU7-U2AF65, side chain contacts (s), water-mediated contacts (w) and main chain contacts (m) were compiled from the structure of the human U2AF65/rU7 complex (26).

RRM1

```
rU7-U2AF65        s s  ww                                            sws s
PUF60-RRM1       CRVYVGSIYYELGEDTIRQAFAPF-----------GPIKSIDMSWDSVTMKHKGFAFVEY 177
U2AF65-RRM1      RRLYVGNIPFGITEEAMMDFFNAQMRLGGLTQAPGNPVLAV------QINQDKNFAFLEF 202
SF3B4-RRM1       ATVYVGGLDEKVSEPLLWELFLQA-----------GPVVNTHMPKDRVTGQHQGYGFVEF
                   :***.:   : *  : : *              .*:            :.:.:.*:*:

                                             m
rU7-U2AF65                              s  m sm
PUF60-RRM1       EVPEAAQLALEQMNSVMLGGRNIKVGRPSN 207
U2AF65-RRM1      RSVDETTQAMA-FDGIIFQGQSLKIRRPHD 231
SF3B4-RRM1       LSEEDADYAIKIMNMIKLYGKPIRVNKASA
                    : :  *:  :: : : *: ::: :
```

RRM2

```
                  w                             w
rU7-U2AF65        s s ss  s                     s   s  s   sss s
PUF60-RRM2       NRIYVASVHQDLSDDDIKSVFEAFGKIK-SCTLARDPTTGKHKGYGFIEYEKAQSSQDAV 283
U2AF65-RRM2      HKLFIGGLPNYLNDDQVKELLTSFGPLK-AFNLVKDSATGLSKGYAFCEYVDINVTDQAI 317
SF3B4-RRM2       ANIFIGNLDPEIDEKLLYDTFSAFGVILQTPKIMRDPDTGNSKGYAFINFASFDASDAAI
                  .:::..:   :.:. : . : :** :  : .: :*  **  ***.* :: . : :: *:

                                  w
rU7-U2AF65                 ss   s m
PUF60-RRM2       SSMNLFDLGGQYLRVGKAVT 303
U2AF65-RRM2      AGLNGMQLGDKKLLVQRASV 337
SF3B4-RRM2       EAMNGQYLCNRPITVSYAFK
                  .:*   * .: : *  *
```

Figure S3 Splicing pattern of PUF60-/U2AF65-dependent reporters in cells overexpressing SF3B4

A, Amino-acid substitutions in SF3B4 in cancer. Data are from the COSMIC database, v. 83 (23). Data could not be corrected for multiple database entries from the same patients or other redundancies (Dave Beare, COSMIC, the Sanger Centre, personal communication). **B,** Coexpression of the *UBE2F* (upper panel) or *OGDH* (middle panel) reporters with human SF3B4 and controls in human embryonic kidney 293 cells. RNA products (to the right) are schematically shown in Figure 1. Immunoblot from transfected cells is in the lower panel.

A

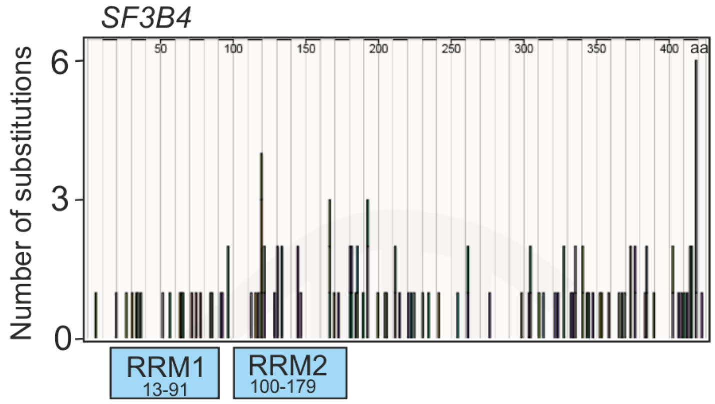

B

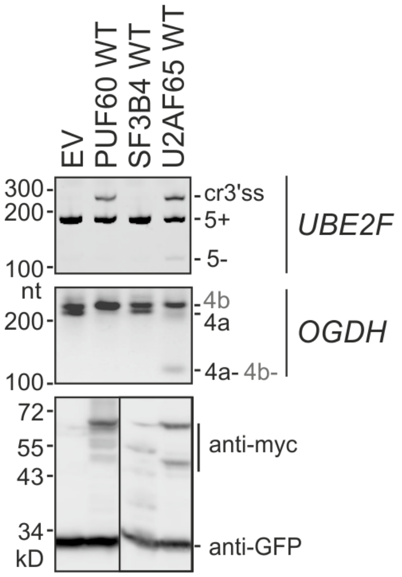

Figure S4 U2AF65 E162K binding to oligoribonucleotides derived from canonical and cryptic 3’ss of *UBE2F* exon 5

A, EMSA with purified recombinant U2AF65 E162K and RNAs indicated at the bottom. The amounts of proteins and RNAs were the same as for the WT (shown in Figure 6A,B). **B,** Fraction of bound RNA with K_d estimates. Hill coefficients were 2.23±0.20 (can) and 1.84±0.21 (cr).

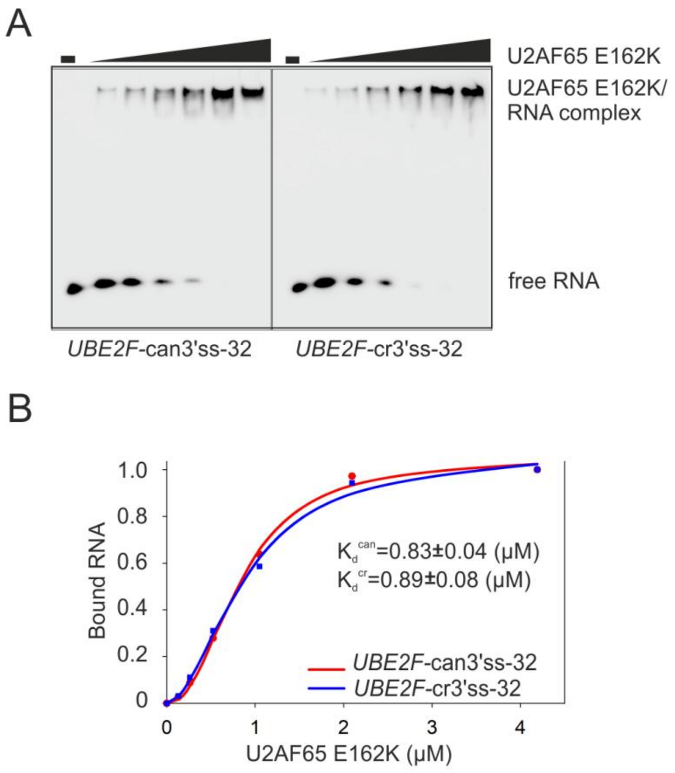

Figure S5 Non-isotopic EMSA with WT and mutated PUF60 and RNA probes derived from competing 3'ss of *UBE2F* exon 5

Substitutions are shown at the top, biotin-labelled RNA probes at the bottom. Their sequences are in Table 1. Concentration of recombinant proteins was 0.19, 0.58 and 1.76 µM.

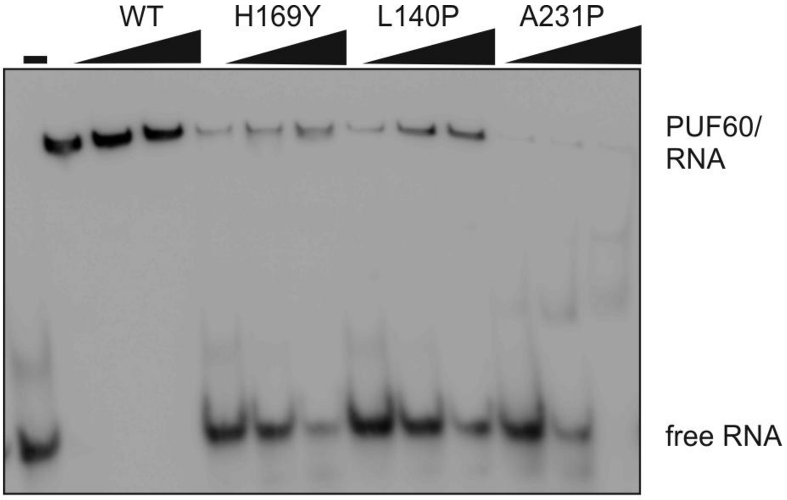

UBE2F-can3'ss-32

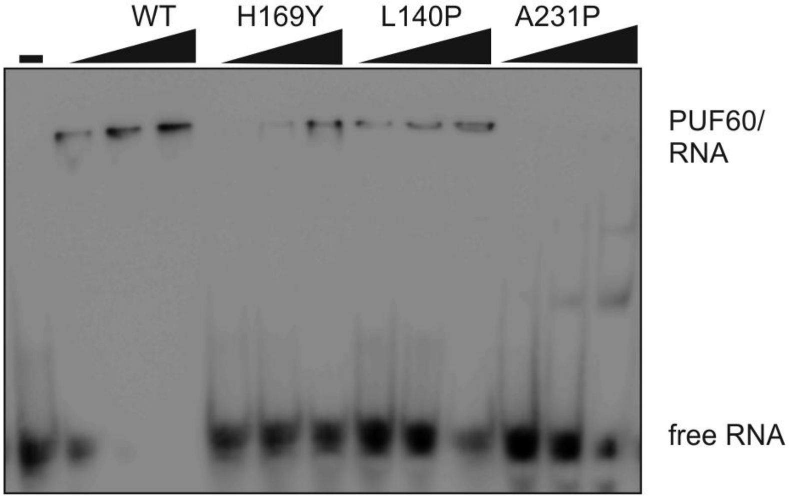

UBE2F-cr3'ss-32

Figure S6 Binding of PUF60 H169Y to *UBE2F*-derived oligoribonucleotides

A, EMSA with purified recombinant PUF60 H169Y and 32-mer RNAs derived from canonical (left panel) and cryptic (right) 3’ss. The amount of proteins and RNAs was the same as for the WT (shown in Figure 7B). **B,** Fraction of bound RNA with approximate K_d estimates (without saturation). The Hill coefficients were 2.4±0.5 (can) and 6.4±2.5 (cr).

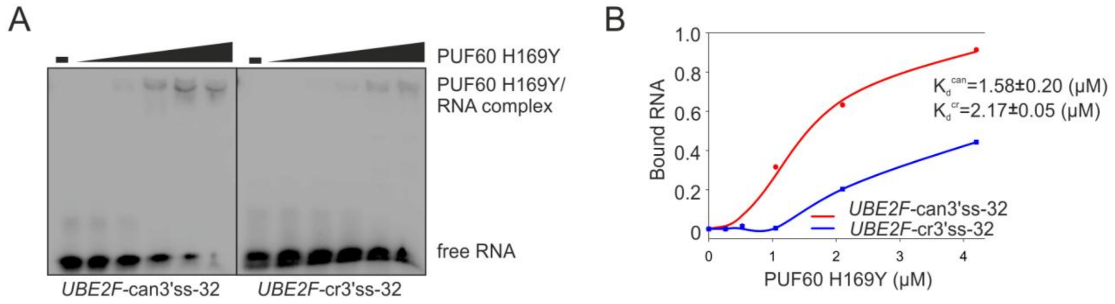

Figure S7 PUF60 L140P and A231P induce protein destabilization and misfolding

Coomassie-stained gel (upper panel) blotted with anti-His antibodies (lower panel) after overnight digestion of the indicated proteins with the TEV protease. The amount of each protein loaded on to the gel was 1.40, 0.46 and 0.14 µg.

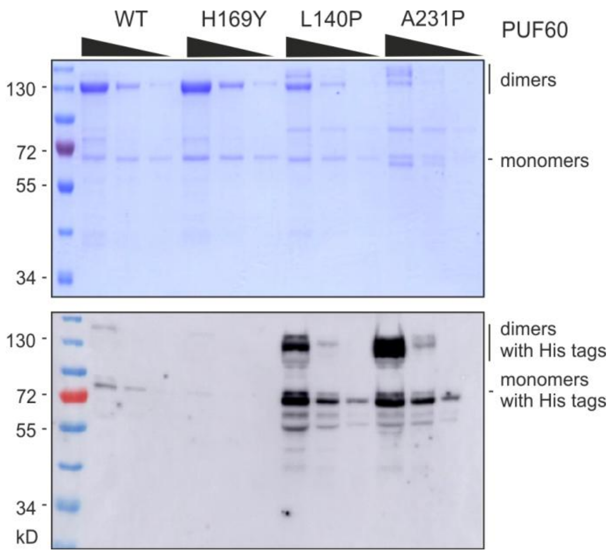

Figure S8 Solubility and stability profiles of wild-type and mutated PUF60 or U2AF65

A, PUF60. **B,** U2AF65. Scores higher than 1 denote highly soluble regions whereas scores smaller than -1 denote poorly soluble regions, as predicted by CamSol (27). Blue/red arrows denote substitutions in regions with potential increased/reduced solubility. Changes in protein stability (bottom right) were predicted using a mutation cut-off scanning matrix (28).

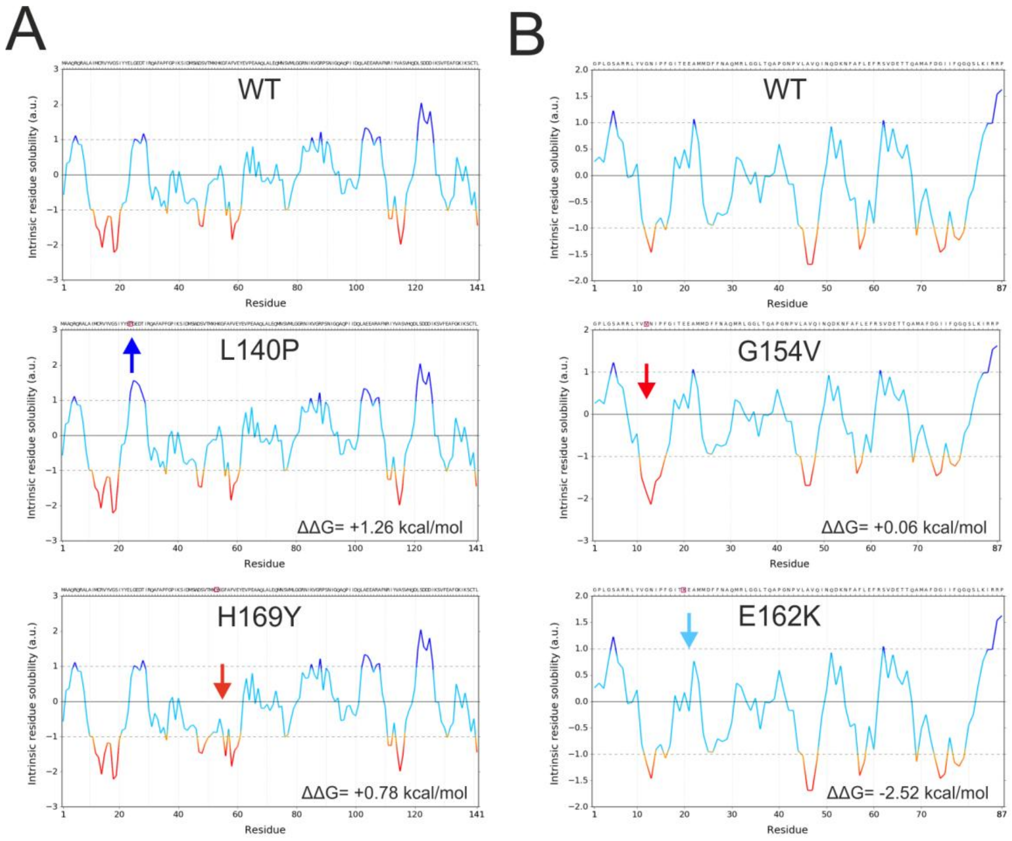

Figure S9 H169Y in the crystal structure of PUF60 RRM1

PDB: 5KW1 (Crichlow et al., unpublished). The protein is shown as cartoon in purple. H169Y and surrounding hydrophobic residues are shown in orange, nitrogen atoms in blue, and oxygen atoms in red. Dotted line denotes a hydrogen bond between H169 and L140.

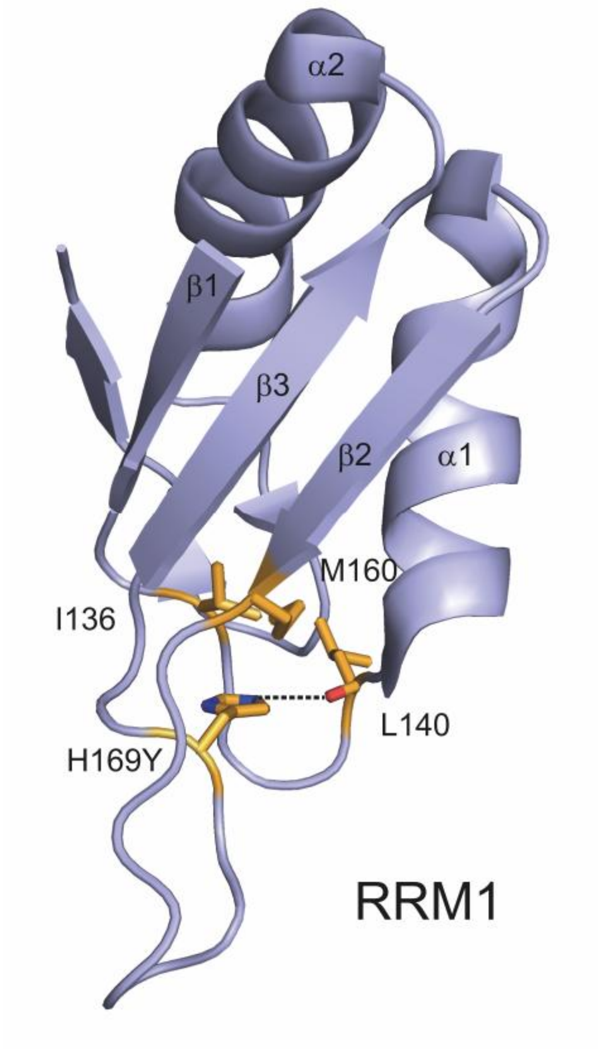

Figure S10 Utilization of cryptic 3'ss of *UBE2F* exon 5 in human tissues

A, Schematics of the *UBE2F* splicing pattern. Exons are shown as boxes, introns as lines, spliced products (shown to the right) as dotted lines. Arrows indicate locations of primers for amplifying canonical (in exon 4 and 6) and non-canonical (across cryptic 3'ss of exon 5 and in exon 6) products. Asterisk denotes a G>A mismatch between the primer and the first position of exon 4a (the sequence is in panel **C**). **B,** RT-PCR products amplified from cDNA samples prepared from human tissues and separated on agarose gels. Amplification primers (Table S2) are schematically shown at the bottom. The cryptic 3'ss of exon 5 could be visualized with primers in exons 4 and 6 only after >33 PCR cycles (not shown). MW, size marker (nt); NC, no target control. Minor species/heteroduplexes are denoted by asterisks. **C,** Sanger sequencing of the two non-canonical RNA products excised from panel B lanes identified to the left.

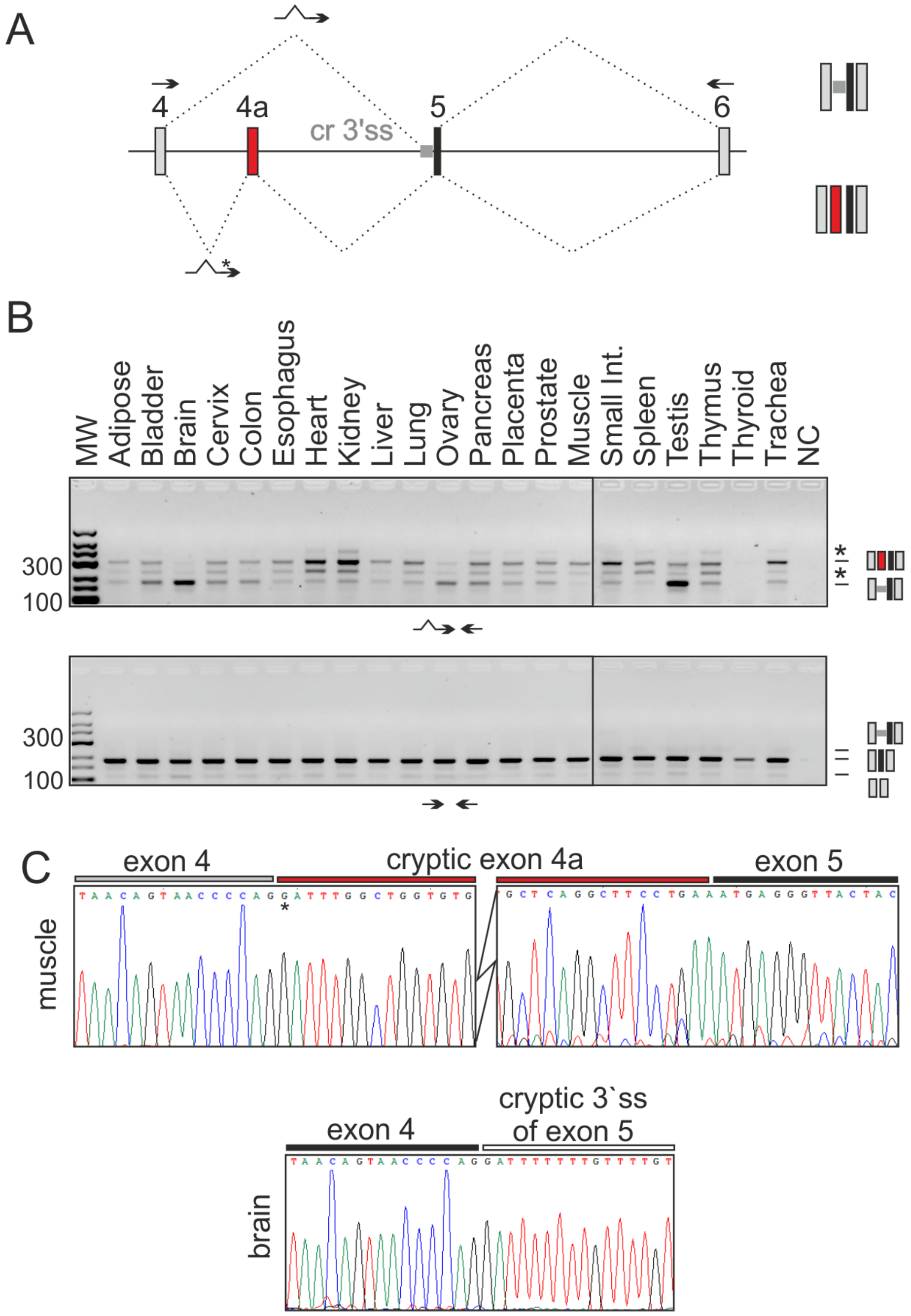

References to Supplemental information

1. Giannakis, M., Hodis, E., Jasmine Mu, X., Yamauchi, M., Rosenbluh, J., Cibulskis, K., Saksena, G., Lawrence, M.S., Qian, Z.R., Nishihara, R. *et al.* (2014) RNF43 is frequently mutated in colorectal and endometrial cancers. *Nat. Genet.*, **46**, 1264-1266.
2. Ellis, M.J., Ding, L., Shen, D., Luo, J., Suman, V.J., Wallis, J.W., Van Tine, B.A., Hoog, J., Goiffon, R.J., Goldstein, T.C. *et al.* (2012) Whole-genome analysis informs breast cancer response to aromatase inhibition. *Nature*, **486**, 353-360.
3. Seshagiri, S., Stawiski, E.W., Durinck, S., Modrusan, Z., Storm, E.E., Conboy, C.B., Chaudhuri, S., Guan, Y., Janakiraman, V., Jaiswal, B.S. *et al.* (2012) Recurrent R-spondin fusions in colon cancer. *Nature*, **488**, 660-664.
4. Waterfall, J.J., Arons, E., Walker, R.L., Pineda, M., Roth, L., Killian, J.K., Abaan, O.D., Davis, S.R., Kreitman, R.J. and Meltzer, P.S. (2014) High prevalence of MAP2K1 mutations in variant and IGHV4-34-expressing hairy-cell leukemias. *Nat. Genet.*, **46**, 8-10.
5. Berger, M.F., Hodis, E., Heffernan, T.P., Deribe, Y.L., Lawrence, M.S., Protopopov, A., Ivanova, E., Watson, I.R., Nickerson, E., Ghosh, P. *et al.* (2012) Melanoma genome sequencing reveals frequent PREX2 mutations. *Nature*, **485**, 502-506.
6. Barbieri, C.E., Baca, S.C., Lawrence, M.S., Demichelis, F., Blattner, M., Theurillat, J.P., White, T.A., Stojanov, P., Van Allen, E., Stransky, N. *et al.* (2012) Exome sequencing identifies recurrent SPOP, FOXA1 and MED12 mutations in prostate cancer. *Nat. Genet.*, **44**, 685-689.
7. Kumar, A., Coleman, I., Morrissey, C., Zhang, X., True, L.D., Gulati, R., Etzioni, R., Bolouri, H., Montgomery, B., White, T. *et al.* (2016) Substantial interindividual and limited intraindividual genomic diversity among tumors from men with metastatic prostate cancer. *Nat. Med.*, **22**, 369-378.
8. Pickering, C.R., Zhou, J.H., Lee, J.J., Drummond, J.A., Peng, S.A., Saade, R.E., Tsai, K.Y., Curry, J.L., Tetzlaff, M.T., Lai, S.Y. *et al.* (2014) Mutational landscape of aggressive cutaneous squamous cell carcinoma. *Clin. Cancer Res.*, **20**, 6582-6592.
9. Giannakis, M., Mu, X.J., Shukla, S.A., Qian, Z.R., Cohen, O., Nishihara, R., Bahl, S., Cao, Y., Amin-Mansour, A., Yamauchi, M. *et al.* (2016) Genomic Correlates of Immune-Cell Infiltrates in Colorectal Carcinoma. *Cell Rep.*, **17**, 1206.
10. Papaemmanuil, E., Gerstung, M., Malcovati, L., Tauro, S., Gundem, G., Van Loo, P., Yoon, C.J., Ellis, P., Wedge, D.C., Pellagatti, A. *et al.* (2013) Clinical and biological implications of driver mutations in myelodysplastic syndromes. *Blood*, **122**, 3616-3627.
11. Papaemmanuil, E., Gerstung, M., Bullinger, L., Gaidzik, V.I., Paschka, P., Roberts, N.D., Potter, N.E., Heuser, M., Thol, F., Bolli, N. *et al.* (2016) Genomic Classification and Prognosis in Acute Myeloid Leukemia. *N. Engl. J. Med.*, **374**, 2209-2221.
12. Guo, G., Sun, X., Chen, C., Wu, S., Huang, P., Li, Z., Dean, M., Huang, Y., Jia, W., Zhou, Q. *et al.* (2013) Whole-genome and whole-exome sequencing of bladder cancer identifies frequent alterations in genes involved in sister chromatid cohesion and segregation. *Nat. Genet.*, **45**, 1459-1463.
13. Nik-Zainal, S., Davies, H., Staaf, J., Ramakrishna, M., Glodzik, D., Zou, X., Martincorena, I., Alexandrov, L.B., Martin, S., Wedge, D.C. *et al.* (2016) Landscape of somatic mutations in 560 breast cancer whole-genome sequences. *Nature*, **534**, 47-54.
14. Quesada, V., Conde, L., Villamor, N., Ordonez, G.R., Jares, P., Bassaganyas, L., Ramsay, A.J., Bea, S., Pinyol, M., Martinez-Trillos, A. *et al.* (2012) Exome sequencing identifies recurrent mutations of the splicing factor SF3B1 gene in chronic lymphocytic leukemia. *Nat. Genet.*, **44**, 47-52.
15. Kataoka, K., Nagata, Y., Kitanaka, A., Shiraishi, Y., Shimamura, T., Yasunaga, J., Totoki, Y., Chiba, K., Sato-Otsubo, A., Nagae, G. *et al.* (2015) Integrated molecular analysis of adult T cell leukemia/lymphoma. *Nat. Genet.*, **47**, 1304-1315.
16. Abaan, O.D., Polley, E.C., Davis, S.R., Zhu, Y.J., Bilke, S., Walker, R.L., Pineda, M., Gindin, Y., Jiang, Y., Reinhold, W.C. *et al.* (2013) The exomes of the NCI-60 panel: a genomic resource for cancer biology and systems pharmacology. *Cancer Res.*, **73**, 4372-4382.

17. Cancer Genome Atlas, N. (2012) Comprehensive molecular characterization of human colon and rectal cancer. *Nature*, **487**, 330-337.
18. Dutton-Regester, K., Kakavand, H., Aoude, L.G., Stark, M.S., Gartside, M.G., Johansson, P., O'Connor, L., Lanagan, C., Tembe, V., Pupo, G.M. *et al.* (2013) Melanomas of unknown primary have a mutation profile consistent with cutaneous sun-exposed melanoma. *Pigment Cell Melanoma Res.*, **26**, 852-860.
19. Van Allen, E.M., Wagle, N., Sucker, A., Treacy, D.J., Johannessen, C.M., Goetz, E.M., Place, C.S., Taylor-Weiner, A., Whittaker, S., Kryukov, G.V. *et al.* (2014) The genetic landscape of clinical resistance to RAF inhibition in metastatic melanoma. *Cancer Discov.*, **4**, 94-109.
20. Hayes, T.F., Benaich, N., Goldie, S.J., Sipila, K., Ames-Draycott, A., Cai, W., Yin, G. and Watt, F.M. (2016) Integrative genomic and functional analysis of human oral squamous cell carcinoma cell lines reveals synergistic effects of FAT1 and CASP8 inactivation. *Cancer Lett.*, **383**, 106-114.
21. Imielinski, M., Berger, A.H., Hammerman, P.S., Hernandez, B., Pugh, T.J., Hodis, E., Cho, J., Suh, J., Capelletti, M., Sivachenko, A. *et al.* (2012) Mapping the hallmarks of lung adenocarcinoma with massively parallel sequencing. *Cell*, **150**, 1107-1120.
22. Dauber, A., Golzio, C., Guenot, C., Jodelka, F.M., Kibaek, M., Kjaergaard, S., Leheup, B., Martinet, D., Nowaczyk, M.J., Rosenfeld, J.A. *et al.* (2013) SCRIB and PUF60 are primary drivers of the multisystemic phenotypes of the 8q24.3 copy-number variant. *Am. J. Hum. Genet.*, **93**, 798-811.
23. Forbes, S.A., Beare, D., Boutselakis, H., Bamford, S., Bindal, N., Tate, J., Cole, C.G., Ward, S., Dawson, E., Ponting, L. *et al.* (2017) COSMIC: somatic cancer genetics at high-resolution. *Nucleic Acids Res.*, **45**, D777-D783.
24. Agrawal, A.A., McLaughlin, K.J., Jenkins, J.L. and Kielkopf, C.L. (2014) Structure-guided U2AF65 variant improves recognition and splicing of a defective pre-mRNA. *Proc. Natl. Acad. Sci. USA*, **111**, 17420-17425.
25. Low, K.J., Ansari, M., Abou Jamra, R., Clarke, A., El Chehadeh, S., FitzPatrick, D.R., Greenslade, M., Henderson, A., Hurst, J., Keller, K. *et al.* (2017) PUF60 variants cause a syndrome of ID, short stature, microcephaly, coloboma, craniofacial, cardiac, renal and spinal features. *Eur. J. Hum. Genet.*, **25**, 552-559.
26. Sickmier, E.A., Frato, K.E., Shen, H., Paranawithana, S.R., Green, M.R. and Kielkopf, C.L. (2006) Structural Basis for Polypyrimidine Tract Recognition by the Essential Pre-mRNA Splicing Factor U2AF65. *Mol. Cell*, **23**, 49-59.
27. Sormanni, P., Aprile, F.A. and Vendruscolo, M. (2015) The CamSol method of rational design of protein mutants with enhanced solubility. *J. Mol. Biol.*, **427**, 478-490.
28. Pires, D.E., Ascher, D.B. and Blundell, T.L. (2014) mCSM: predicting the effects of mutations in proteins using graph-based signatures. *Bioinformatics*, **30**, 335-342.
